# 2,1,3-Benzothiadiazole-5,6-Dicarboxylic Imide – A Versatile Building Block for Additive- and Annealing-Free Processing of Organic Solar Cells with Efficiencies Exceeding 8%

**DOI:** 10.1002/adma.201404858

**Published:** 2014-12-15

**Authors:** Christian B Nielsen, Raja Shahid Ashraf, Neil D Treat, Bob C Schroeder, Jenny E Donaghey, Andrew J P White, Natalie Stingelin, Iain McCulloch

**Affiliations:** Department of Chemistry and Centre for Plastic Electronics, Imperial College LondonLondon, SW7 2AZ, UK; Department of Materials and Centre for Plastic Electronics, Imperial College LondonLondon, SW7 2AZ, UK; Physical Sciences and Engineering Division, King Abdullah University of Science and Technology (KAUST)Thuwal, 23955-6900, Saudi Arabia

**Keywords:** donor–acceptor polymers, organic photovoltaics, organic electronics

The development of organic semiconductors for organic photo­voltaic (OPV) applications has advanced rapidly in recent years. While the electron acceptor of choice is still predominantly a fullerene derivative such as [6,6]-phenyl-C71-butyric acid methyl ester (PC_71_BM), an immense array of different light-absorbing electron-donor materials has been reported and encouragingly device efficiencies for single junction cells are approaching 10%.^1^,^2^ In this context, it is worth noting that all the highest performing OPV devices have been achieved with the use of solvent processing additives, additional organic extraction layers, or similar device engineering efforts.^3^–^8^ Although important for driving the field of organic photovoltaics forward, significant manufacturing challenges are associated with the increased complexity of the photovoltaic device. Therefore, it is still crucial to pursue and further improve the synthetic design and development of organic light absorbing materials, which have yet to surpass the 8% efficiency mark in a standard solar cell configuration without solvent additives or additional organic extraction layers.

Polymeric electron donor materials are typically designed using a push–pull strategy where electron-rich and electron-deficient π-conjugated motifs alternate along the polymer backbone, whereupon molecular orbital hybridization results in a narrow band gap material with good spectral overlap with the solar spectrum. One of the most widely used electron-deficient motifs is the 2,1,3-benzothiadiazole (BT) (**Figure** **1**) successfully incorporated into numerous polymers, including for example poly[*N*-9′-heptadecanyl-2,7-carbazole-*alt*-5,5-(4′,7′-di-2-thienyl-2′,1′,3′-benzothiadiazole)] (PCDTBT)^9^ and poly[2,6-(4,4-bis-(2-ethylhexyl)-4H-cyclopenta[2,1-b;3,4-b′]-dithiophene)-*alt*-4,7-(2,1,3-benzothiadiazole)] (PCPDTBT),^10^ which are two of the most studied push–pull type materials in OPV research. Building on the BT motif, the free 5- and 6-positions have proven to be useful locations for attaching either electron-donating or electron-withdrawing substituents in order to adjust the frontier molecular orbitals in the resulting copolymers as well as to often enhance solubility.^11^ This approach is illustrated in Figure 1 where the dialkoxy-functionalized BT (O2BT) moiety represents a weaker electron-accepting, and more soluble moiety than the parent BT moiety,^12^ while the 5,6-difluoro-2,1,3-benzothia­diazole (F2BT) system correspondingly represents a stronger electron-accepting unit. In particular, fluorination of the BT moiety has led to many new high-performing OPV materials.^7^,^13^–^17^ Introduction of the highly electronegative fluorine atom(s) is generally found to stabilize both the lowest unoccupied molecular orbital (LUMO) and the highest occupied molecular orbital (HOMO), thus affording an increase in open-circuit voltage (*V*_oc_), while other OPV device parameters are largely unaffected in cases where there is sufficient energetic offset for electron transfer to the fullerene. Recently, a cyclic imide has been attached to the BT unit to afford 2,1,3-benzothiadiazole-5,6-dicarboxylic imide (BTI) as illustrated in Figure 1.^18^,^19^

**Figure 1:**
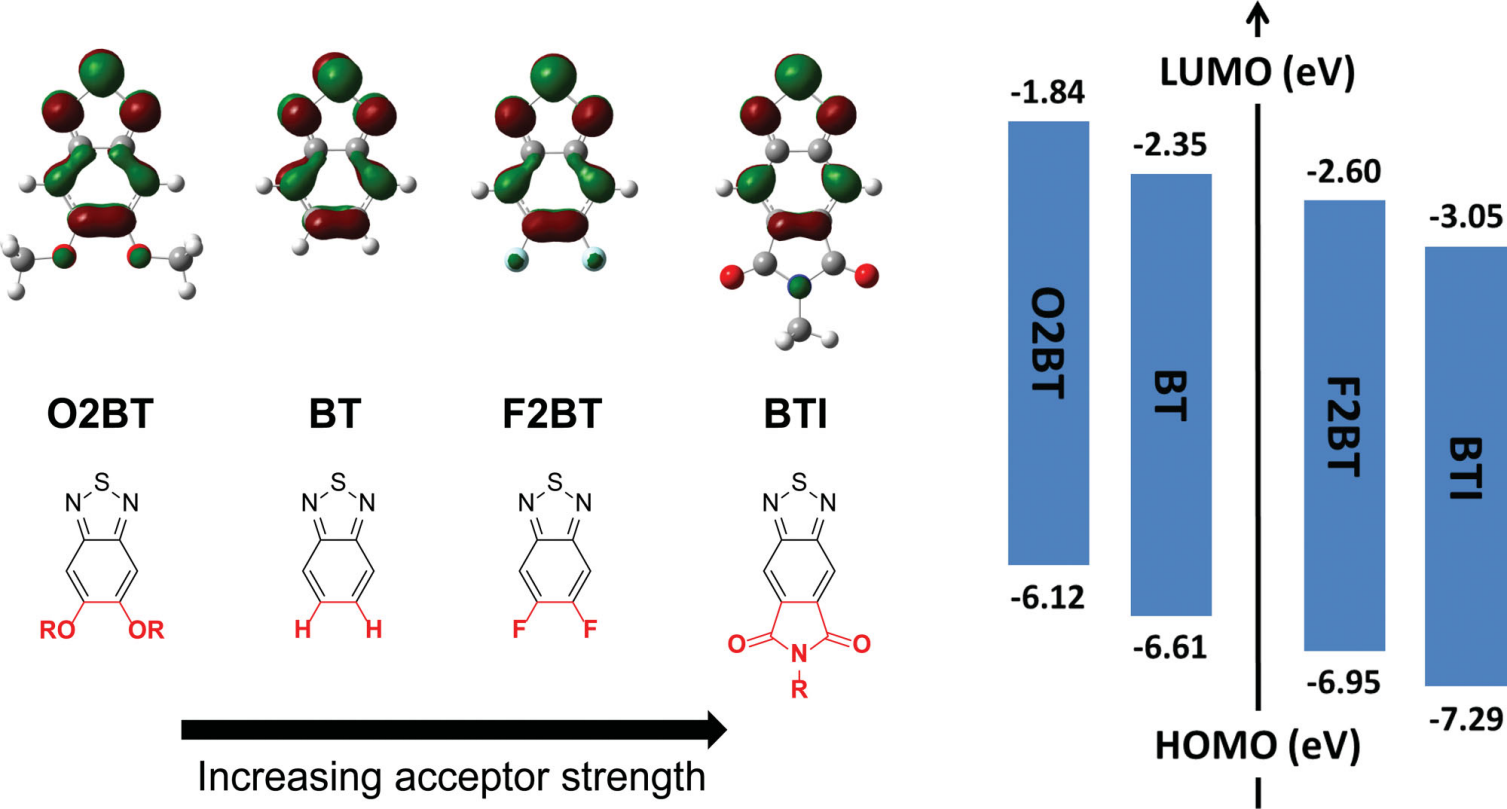
Examples of chemical modifications of the 2,1,3-benzothiadiazole system in the 5- and 6-positions with theoretical HOMO- and LUMO-energy levels (right) and spatial LUMO distributions (top); all data obtained using Gaussian at the B3LYP/6–31G* level of theory.

Comparing these BT derivatives from a semi-empirical perspective, the HOMO–LUMO gaps are found to be nearly identical for the four compounds (4.24–4.35 eV), while there is a significant lowering of the LUMO energy level across the series (Figure 1, right hand side) indicating that incorporation of the cyclic imide results in a stronger electron-accepting unit than what is achieved by fluorination of the BT unit. The concurrent lowering of the HOMO energy level should simultaneously result in a further increase in the *V*_oc_ compared to photo­active materials incorporating the F2BT unit. The calculations furthermore indicate that the imide has little influence on the molecular orbital distributions, which are nearly identical for the four molecules (illustrated for the LUMOs in Figure 1).

Importantly, from a synthetic point of view, the imide provides a site for alkyl substitution through its nitrogen atom. While the alkyl chain density needed to induce sufficient solubility and processability for BT and F2BT containing copolymers can only be introduced via the electron-rich co-monomer, BTI containing copolymers can be solubilized by a combination of alkyl chains attached to the electron-rich and the electron-deficient co-monomers. This added degree of freedom in the synthetic design allows for a more thorough investigation of alkyl chain substitution patterns without compromising solubility and/or molecular weight. Consequently, as the alkyl substitution pattern frequently has been found to impact the polymer/fullerene microstructure greatly,^20^,^21^ a more thorough optimization of OPV device performance can be realized through synthetic design and less emphasis needs to be directed towards sensitive processing parameters such as solvent mixtures, solvent additives, and solvent- and temperature-annealing steps.^10^,^22^–^24^

As illustrated in **Scheme** **1**, polymerizations were carried out with the dithienyl flanked BTI monomer and benzo[1,2-b:3,4-b′:5,6-d′]trithiophene (BTT).^25^ The fully fused and planar aromatic structure of BTT was chosen as the co-monomer to facilitate good charge transport.^26^ Furthermore, the moderately electron-rich BTT unit was chosen to afford a polymer with a relatively narrow band gap and a deep HOMO level to give high *J*_sc_ and *V*_oc_ values. Reassuringly, the BTT co-monomer has previously shown good promise in OPV devices.^27^,^28^ Two different BTT monomers with either a linear *n*-hexadecyl (C16) side-chain or a branched 1-nonyldecyl (C9C10) side-chain were distannylated according to literature procedures.^25^,^27^ In a Pd-catalyzed Stille-type polymerization, the two distannylated BTT monomers were separately reacted with the dibrominated BTI monomer with a branched 2-decyltetradecyl (C10C14) side-chain to afford the BTT-*co*-BTI polymers BBTI-1 and BBTI-2 with a linear C16 and a branched C9C10 chain on the BTT unit, respectively. BBTI-1 was obtained with a molecular weight (*M*_n_) of 64 kDa and a polydispersity index (PDI) of 1.99, while BBTI-2 had an *M*_n_ value of 75 kDa (PDI 2.11). Both polymers are fully soluble in chlorinated solvents as well as in environmentally friendlier nonchlorinated solvents such as toluene and xylene. The two polymers display high thermal stability with 5% weight loss above 460 °C (Figure S9, Supporting Information). No discernible thermal events were observed for either polymer when analyzed by differential scanning calorimetry in the temperature range 0–300 °C (Figure S10, Supporting Information).

**Scheme 1:**
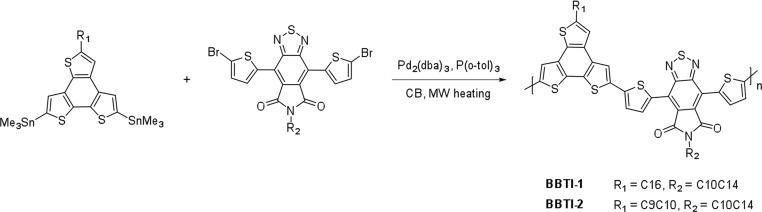
Synthetic route towards BTI-based polymers.

The dithienyl flanked BTI monomer with a short *n*-butyl side-chain was synthesized for model studies and a single crystal was successfully analyzed by X-ray diffraction (XRD). To understand the effect of forming the cyclic imide, the noncyclized BT-diester was also crystallized and studied by XRD and the two molecular conformations obtained from the crystal structures are depicted in **Figure** **2**. While the flanking thiophenes of the diester are disordered showing two different orientations, the thiophene units in the imide are free of disorder (see the Supporting Information). We furthermore note that the dihedral angles (*θ*_A_ and *θ*_B_) are slightly smaller for the imide (ca. 41.2°, 40.3°, 38.5°, and 37.3° for the four bonds to the thiophene units across the two independent molecules) than for the non-cyclized diester (ca. 47.8° and 41.6°) as could be expected when reducing the steric hindrance between adjacent rings upon cyclization, though the disorder in the rings for the latter makes this uncertain. In comparison, other closely related BT derivatives such as dithieno-BT and dithieno-F2BT adopt a completely coplanar orientation in the solid state.^29^ Although the torsional twist observed for the BTI monomer in the solid state could potentially reduce the effective π-conjugation along the polymer backbone, we note that variable temperature NMR experiments (Figure S8, Supporting Information) show no sign of hindered rotational freedom of the flanking thiophene rings even at 183 K where *kT* is 1.5 kJ mol^−1^. In agreement with XRD data, semi-empirical calculations using Gaussian at the B3LYP/6–31G* level predict the conformation of the dithienyl flanked BTI monomer in Figure 2 to be the most energetically favorable conformation.

**Figure 2:**
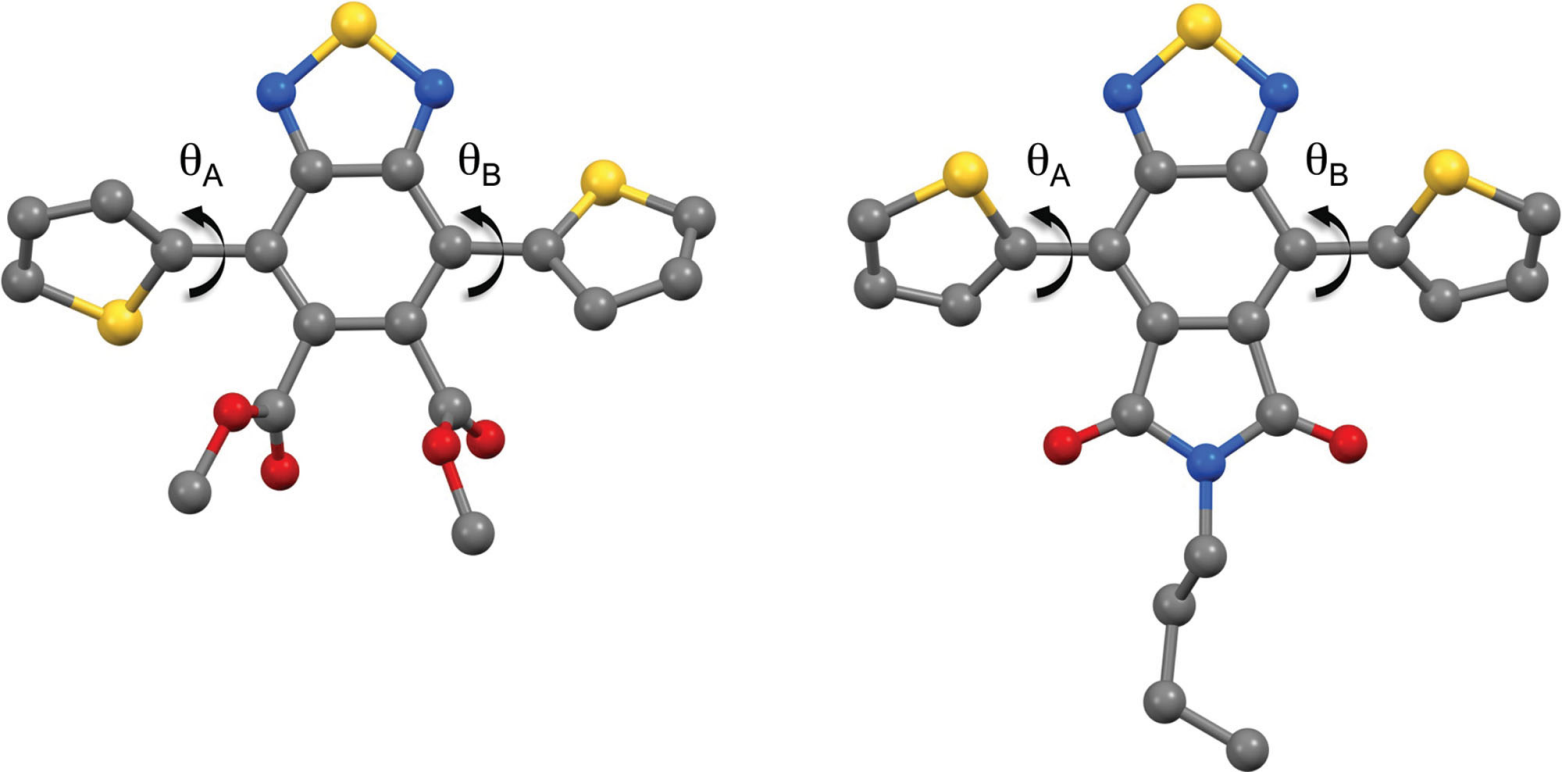
Crystal structures of dimethyl 4,7-di(2-thienyl)-2,1,3-benzothiadiazole-5,6-dicarboxylate (left) and *N*-butyl-4,7-di(2-thienyl)-2,1,3-benzothiadiazole-5,6-dicarboxylic imide (right) with color coded atoms: C (grey), N (blue), O (red), and S (yellow); all hydrogen atoms have been omitted for clarity. The cyclic imide crystallized with two independent molecules that have nearly identical molecular conformations (see the Supporting Information for more details).

The optical properties of BBTI-1 and BBTI-2 were measured by UV–vis spectroscopy in chlorobenzene (CB) solution and in the solid state as thin films spin-cast from CB solution (Figure S11, Supporting Information). In solution, BBTI-1 has an absorption maximum at 705 nm, while BBTI-2 is slightly blue-shifted with a maximum at 700 nm; the molar extinction coefficients (ε_max_) were found to be 4.2 × 10^4^ M^−1^ cm^−1^ for both polymers. When going from solution to the solid state, both polymers are red-shifted approximately 20 nm and the slightly red-shifted absorption maximum of BBTI-1 (722 nm) relative to BBTI-2 (716 nm) is thus also observed in the solid state. It appears that BBTI-1 with the linear side-chain on the BTT unit adopts a slightly more coplanar orientation in the solid state than BBTI-2 with the branched alkyl chain. This is supported by the difference in absorption coefficients in the solid state, which are found to be 2.0 × 10^5^ cm^−1^ for BBTI-1 and 1.5 × 10^5^ cm^−1^ for BBTI-2; values which are comparable to that of poly(3-alkylthiophene).^30^

Oxidative cyclic voltammetry was used to investigate the electrochemical properties of the polymers. Both polymers show reversible oxidative behavior (Figure S12, Supporting Information) with onsets of oxidation found at 0.34 and 0.43 V (vs Fc/Fc^+^), respectively, for BBTI-1 and BBTI-2. From the onsets of oxidation, the HOMO energy levels are estimated at −5.2 eV for BBTI-1 and −5.3 eV for BBTI-2. Corroborating the optical properties, the higher-lying HOMO level of BBTI-1 relative to BBTI-2 is in agreement with a more coplanar orientation of the polymer backbone in the thin film of BBTI-1 than in the thin film of BBTI-2. We note that a HOMO level around −5.2 eV and a band gap of roughly 1.5 eV are very encouraging characteristics for an OPV donor material designed to be used with fullerene acceptors due to a sufficient LUMO–LUMO offset and a good spectral overlap with the solar flux.^31^

The photovoltaic properties of BBTI-1 and BBTI-2 were examined in an inverted OPV device architecture with PC_71_BM as the electron acceptor material. Organic photovoltaic device data concerning conventional device architectures are included in Table S1, Supporting Information. The *J–V* curves are presented in **Figure** **3** and the data are summarized in **Table** **1**. The photoactive layers comprised a 1:2 weight ratio of polymer to fullerene and were firstly solution cast from neat *o*-dichlorobenzene (ODCB). Using these conditions, BBTI-1 afforded a very high power conversion efficiency (PCE) of 8.3% owing to a high short-circuit current (*J*_sc_) of 16.45 mA cm^−2^, a high *V*_oc_ of 0.80 V, and a respectable fill factor (FF) of 0.63. BBTI-2, on the other hand, afforded an inferior OPV device with a PCE of 3.3%. Although the *V*_oc_ was significantly higher in accordance with the difference in HOMO values, the efficiency was compromised by a low *J*_sc_ value. Interestingly, when 1,8-diiodooctane (DIO) was employed as a solvent additive, the performance of BBTI-1 significantly decreased (4.8% PCE) due to a loss in current, voltage, and fill factor (Table1), whereas the PCE of BBTI-2 was nearly doubled to 6.0% as a consequence of a greatly improved *J*_sc_ (11.00 mA cm^−2^) and a significantly higher fill factor of 0.67. While the data reported in Table1 are for the best OPV devices, average values obtained from six devices were likewise determined for BBTI-1 (without additive) giving an average PCE of 8.1%.

**Table 1:** Photovoltaic device parameters for inverted configuration solar cells with BBTI-1 and BBTI-2^a^

	Additive	*J*_sc_ [mA cm^−2^]	*V*_oc_ [V]	FF	PCE [%]
BBTI-1	None	16.45	0.80	0.63	8.3
BBTI-1	3% DIO	14.11	0.74	0.46	4.8
BBTI-2	None	7.12	0.85	0.54	3.3
BBTI-2	3% DIO	11.00	0.81	0.67	6.0

a Device configuration: ITO/ZnO/polymer:PC_71_BM(1:2)/MoO_3_/Ag.

**Figure 3:**
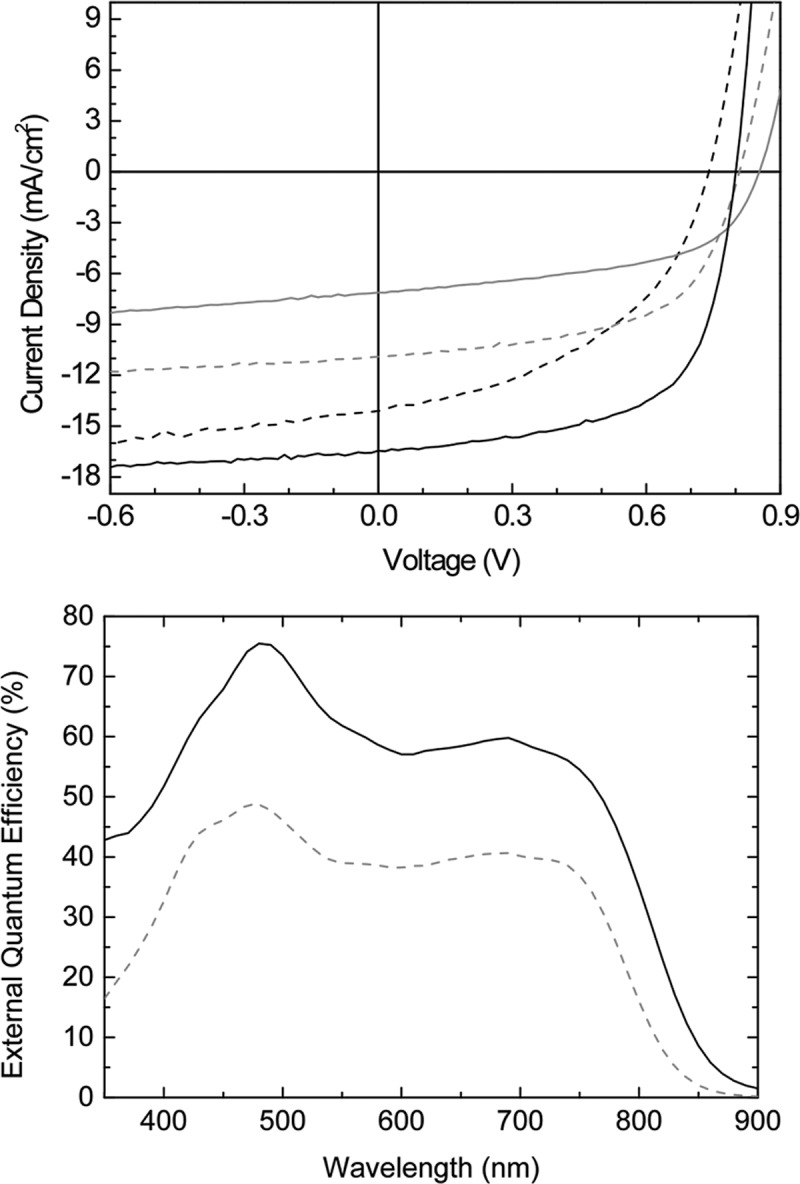
*J*–*V* (left) and EQE (right) curves of OPV devices with BBTI-1 (black lines) and BBTI-2 (gray lines) in the photoactive layer with (dashed lines) and without (full lines) DIO additive.

The external quantum efficiency (EQE) curves for the best devices with BBTI-1 and BBTI-2 are depicted in Figure 3. Both devices show a broad spectral response in the 400–800 nm region, with peak EQE values at 75% for BBTI-1 and 49% for BBTI-2. The maxima are observed around 480 nm where both the polymers and PC_71_BM absorb, but significant photocurrent generation from the polymers is also evident from the high EQE response (≈60% for BBTI-1 and ≈40% for BBTI-2) in the 600–750 nm region. Integration of the EQE spectra with the AM1.5G solar spectrum affords predicted *J*_sc_ values of 16.48 mA cm^−2^ for BBTI-1 and 10.67 mA cm^−2^ for BBTI-2; values which are in excellent agreement with the data reported in Table1.

To the best of our knowledge, the PCE of 8.3% for BBTI-1 achieved by simple solution processing from neat ODCB is on a par with the best OPV performance reported to date in a standard device configuration (conventional or inverted) without the use of any solvent additives or annealing steps.^32^–^37^ We believe that the aspect of simple processing conditions and thus a robust and reproducible protocol for device fabrication is an important consideration when designing novel photoactive materials and obviously of great interest and significance for the commercialization of organic solar cells.

It is well-established that a strong correlation exists between the OPV device performance and the bulk heterojunction (BHJ) blend morphology.^24^ From the XRD diffractograms of thick drop-cast films of neat BBTI-1 and BBTI-2 (Figure S13, Supporting Information), it is evident that BBTI-1 with the linear C16 chain shows a much higher degree of edge-on crystallinity than BBTI-2 with the branched C9C10 chain. BBTI-1 orients predominantly edge-on relative to the substrate with a lamellar repeat distance of 23.4 Å, while BBTI-2 is much less ordered with few edge-on crystallites and also few face-on crystallites with a π-stacking distance of approximately 3.6 Å. This is in full agreement with the observation that BBTI-1 tends to aggregate more in solution than BBTI-2, which will result in π-stacked plate-like aggregates in solution and hence a predominant edge-on orientation on the substrate.^38^ Considering the BHJ blends, the surface morphology was examined with atomic force microscopy (AFM) as illustrated in **Figure** **4**. While the BBTI-1 blend without additives has a finely intermixed phase separation judged from its surface morphology, introduction of the DIO additive clearly disrupts the homogeneity of this phase separation (i.e., areas with both coarser and finer phase separation), which is likely to account for the drop in PCE from 8.3% to 4.8%. The AFM height histograms show that the average domain size increases from roughly 8 nm to 11 nm upon introduction of DIO, which again corroborates the observed drop in efficiency when considering typical exciton diffusion lengths to be 5–10 nm.^39^,^40^ Compared to BBTI-1, the photoactive blend with BBTI-2 (no additive) has a much coarser phase separation, which could explain the poor device performance (PCE of 3.3%). For BBTI-2, the DIO additive clearly prevents the coarse phase separation (hence the improved device efficiency of 6.0%), although the blend morphology judged from AFM still looks quite different from the higher performing BBTI-1 devices. For BBTI-2, the height histograms clearly emphasize the coarse and unfavorable phase separation for the blend without solvent additive, while DIO reduces the domain size noticeably to a length scale comparable to the excitons diffusion length. High-resolution scanning electron microscopy (SEM) (Figure S17, Supporting Information) correspondingly indicated that the blend with BBTI-1 has a very homogeneous phase separation, while addition of DIO gives rise to a much more irregular surface morphology. For BBTI-2, SEM confirms the coarse and detrimental blend morphology, while it is also clear that introduction of DIO significantly improves the phase separation.

**Figure 4:**
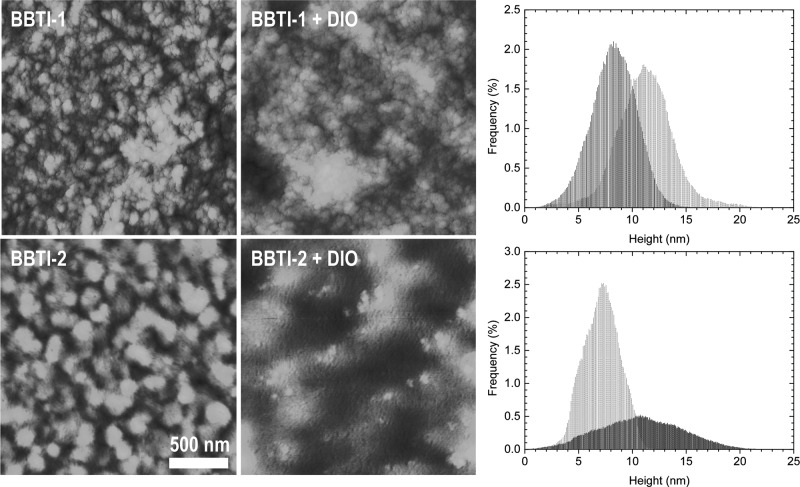
Atomic force microscopy images (2 μm × 2 μm) showing the topography of blend films (polymer:PC_71_BM in a 1:2 weight ratio cast from *o*-dichlorobenzene solution) for BBTI-1 (top) and BBTI-2 (bottom) without solvent additive (left) and with 3% 1,8-diiodooctane (middle); the right panels shows the height histograms for BBTI-1 (top) and BBTI-2 (bottom) without additive (black) and with additive (gray).

Through our synthetic design rationale, the planar and fused BTT moiety was employed as an electron-rich co-monomer in conjunction with the electron-deficient BTI unit to create a highly absorptive π-conjugated polymer with an ideal band gap of roughly 1.5 eV and optimum frontier energy levels allowing for efficient charge transfer to a fullerene and a high open circuit voltage. Importantly, both the BTT unit and the BTI unit have easily accessible sites for alkyl chain attachment, which facilitates rapid optimization of alkyl chain substitution pattern in order to achieve a soluble and processable high molecular weight polymeric material with optimum phase separation with fullerene acceptors. Judging from the HOMO and LUMO distributions calculated semi-empirically (Figure S20, Supporting Information), both the alkyl chain on the BTT unit and the alkyl chain on the BTI unit are positioned away from the frontier orbital distributions. The HOMO is delocalized along the π-conjugated backbone allowing for good hole transport, while the LUMO is localized on the electron-deficient BTI unit. Focusing on the LUMO, we note that the orbital distribution is centered around the benzene and the thiadiazole rings and not on the cyclic imide. We believe this to be an important aspect of our synthetic design, because not only the alkyl chain on the electron-rich component but also the alkyl chain on the electron-deficient component is spatially separated from the LUMO distribution, the photoactive polymer tolerates branched alkyl chains (often needed for enhanced solubility and good BHJ blend intermixing) on both co-monomers. This is in contrast to for example TPD-based polymers, where recent work has highlighted a drastic decrease in OPV performance when branched alkyl chains are attached to the electron-deficient co-monomer.^41^

In conclusion, we have introduced a new versatile polymer, BBTI, which has ideal frontier energy levels for organic photovoltaics and can be easily synthesized with the optimum alkyl chain substitution pattern. This in turn generates an excellent blend morphology with the PC_71_BM acceptor without the need for solvent additives or thermal or solvent-assisted annealing steps during the device fabrication. As a consequence, BBTI can be solution cast in a blend with PC_71_BM from a single solvent system to reproducibly afford organic solar cells with power conversion efficiencies above 8% with maximum efficiencies reaching 8.3%.

CCDC 1017114 and 1017115 contain the Supporting Information crystallographic data for this paper. These data can be obtained free of charge from The Cambridge Crystallographic Data Centre via www.ccdc.cam.ac.uk/data_request/cif.

## Supporting Information

As a service to our authors and readers, this journal provides supporting information supplied by the authors. Such materials are peer reviewed and may be re-organized for online delivery, but are not copy-edited or typeset. Technical support issues arising from supporting information (other than missing files) should be addressed to the authors.
