# Supplementary material for: 2,1,3-Benzothiadiazole-5,6-Dicarboxylic Imide – A Versatile Building Block for Additive- and Annealing-Free Processing of Organic Solar Cells with Efficiencies Exceeding 8%
Source: Adv Mater. 2014 Dec 15;27(5):948–53. doi: 10.1002/adma.201404858 (PMC4365755; doi:10.1002/adma.201404858)
Supplement: Supplementary file 1 — Supplementary [file adma0027-0948-sd1.pdf]

Copyright WILEY-VCH Verlag GmbH & Co. KGaA, 69469 Weinheim, Germany, 2014.

ADVANCED MATERIALS

Supporting Information

for *Adv. Mater.,* DOI: 10.1002/adma.201404858

2,1,3-Benzothiadiazole-5,6-Dicarboxylic Imide – A Versatile Building Block for Additive- and Annealing-Free Processing of Organic Solar Cells with Efficiencies Exceeding 8%

Christian B. Nielsen, Raja Shahid Ashraf,* Neil D. Treat, Bob C. Schroeder, Jenny E. Donaghey, Andrew J. P. White, Natalie Stingelin, and Iain McCulloch*

Copyright WILEY-VCH Verlag GmbH & Co. KGaA, 69469 Weinheim, Germany, 2013.

Supporting Information

2,1,3-Benzothiadiazole-5,6-Dicarboxylic Imide – A Versatile Building Block for Additive and Annealing Free Processing of Organic Solar Cells with Efficiencies Exceeding 8%

Christian B. Nielsen, Raja Shahid Ashraf,* Neil D. Treat, Bob C. Schroeder, Jenny E. Donaghey, Andrew J. P. White, Natalie Stingelin, and Iain McCulloch*

Materials and Methods	S2
Experimental Details	S3
NMR spectra of monomers	S6
NMR spectra of polymers	S11
Variable-temperature NMR spectra	S12
TGA traces of polymers	S13
DSC traces of polymers	S13
UV-vis and temperature-dependent UV-vis of polymers	S14
CV scans of polymers	S15
XRD spectra of polymers	S15
Conventional configuration OPV data	S16
AFM micrographs of neat polymers and blends	S16
TEM micrographs of blends	S19
Transmission optical micrographs	S20
Calculated HOMO and LUMO distributions	S22
Crystallographic details	S23

Materials and Methods

All chemicals were purchased from commercial suppliers and used as received unless otherwise specified. Column chromatography was carried out with silica gel for flash chromatography from VWR Scientific. Microwave experiments were performed in a Biotage Initiator V 2.3. ^{1}H and ^{13}C NMR spectra were recorded on a Bruker Model 400 spectrometer. A custom-build Shimadzu SEC system was used to fractionate the polymers with chlorobenzene at 80°C as the eluent. The system comprises a DGU-20A3 degasser, an LC-20A pump, a CTO-20A column oven, an Agilent PLgel 10µm MIXED-D column and a SPD-20A UV detector. Number-average (M_n) and weight-average (M_w) molecular weights were determined with an Agilent Technologies 1200 series GPC in chlorobenzene at 80°C, using two PL mixed B columns in series, and calibrated against narrow weight-average dispersity (D_w < 1.10) polystyrene standards.

The thermal stability of the polymers was analyzed by thermogravimetric analysis (TGA) using a TA Instruments Q50 under a continuous nitrogen purge of 60 mL/min. The samples were heated from room temperature to 600°C with a uniform heating rate of 10°C/min. Differential scanning calorimetry (DSC) was carried out with a TA Instruments DSC Q20. UV−vis absorption spectra were recorded on a UV-1601 Shimadzu UV−vis spectrometer. Photoelectron spectroscopy in air (PESA) measurements were recorded with a Riken Keiki Model AC-2 PESA spectrometer with a power setting of 5 nW and a power number of 0.5. Cyclic voltammetry was performed with a standard three-electrode setup with a Pt-mesh counter electrode and an Ag/Ag^{+} reference electrode calibrated against Fc/Fc^{+} using an Autolab PGSTAT101 potentiostat. The measurements were carried out using spin-cast films (from chlorobenzene solutions at 5 mg/ml) on ITO-coated glass substrates with 0.1M tetrabutylammonium hexafluorophosphate in deoxygenated acetonitrile as supporting electrolyte; scan rate was 50 mV/s. HOMO energy values were obtained using the following equation: $E_{HOMO} = -(E_{ox} - E_{Fc} + 4.88)$ eV.

Specular X-ray diffraction (XRD) was carried out using a PANalytical X’Pert PRO MRD diffractometer equipped with a nickel-filtered Cu-$K_{\alpha 1}$ beam and X’Celerator detector, using current I = 40 mA and accelerating voltage U = 40 kV. Atomic force microscopy (AFM) was carried out using a Dimension 3100 atomic force microscope in close contact (tapping) mode. For the scanning electron microscopy (SEM) experiments, a Carl Zeiss Auriga 40 High resolution Field Emission Scanning Electron Microscope operating at 5 kV was used to analysis the samples and an InLens detector was used to collect the Secondary Electron signals from the surfaces of the samples.

BHJ solar cells were fabricated with an inverted (ITO/ZnO/polymer:$PC_{71}BM/MoO_3$/Ag) configuration and tested under simulated 100 mW/cm^2 AM1.5G illumination. Devices were in all cases prepared with a polymer:$PC_{71}BM$ blend ratio of 1:2 and solution processed from *o*-dichlorobenzene.The pixel size, defined by the spatial overlap of the anode and cathode, was 0.045 cm^2. The device characteristics were obtained using a xenon lamp with AM1.5G filters and 100 mW/cm^2 illumination (Oriel Instruments). Short circuit currents under AM1.5G conditions were obtained from the spectral response and convolution with the solar spectrum, measured with a Keithley source meter. Spectral response was measured under operation conditions using bias light from a 532 nm solid state laser (Edmund Optics). Monochromatic light from a 100 W tungsten halogen lamp in combination with monochromator (Oriel, Cornerstone 130) was modulated with a mechanical chopper. The response was recorded as the voltage over a 50 Ω resistance, using a lock-in amplifier (Stanford research Systems SR830). A calibrated Si cell was used as reference. All the device measurements were carried out behind a quartz window in a nitrogen filled container.

Experimental Details

Dimethyl 4,7-di(2-thienyl)-2,1,3-benzothiadiazole-5,6-dicarboxylate (2)

A solution of **1**[1] (2.26 g, 7.37 mmol) and dimethyl acetylenedicarboxylate (3.6 mL, 29 mmol) in anhydrous toluene (50 mL) was heated at reflux overnight. The reaction mixture was concentrated *in vacuo* and the title compound was subsequently obtained by column chromatography (silica, pentane/dichloromethane) as a yellow solid (2.48 g, 5.95 mmol, 81% yield).

^{1}H NMR (400 MHz, $CDCl_3$): δ 7.60 (dd, J = 5.1, 1.2 Hz, 1H), 7.43 (dd, J = 3.6, 1.2 Hz, 1H), 7.20 (dd, J = 5.1, 3.6 Hz, 1H), 3.76 (s, 3H).

^{13}C NMR (100 MHz, $CDCl_3$): δ 168.17, 153.72, 135.19, 132.18, 129.86, 129.03, 127.39, 126.35, 53.17.

4,7-Di(2-thienyl)-2,1,3-benzothiadiazole-5,6-dicarboxylic acid (3)

A solution of **2** (2.85 g, 6.84 mmol) and sodium hydroxide (4.59 g, 115 mmol) in ethanol (150 mL) was heated at reflux overnight. The reaction mixture was concentrated *in vacuo*, redissolved in water (200 mL) and acidified with concentrated hydrochloric acid to afford the title compound as a yellow solid that was isolated by filtration (2.63 g, 6.77 mmol, 99% yield).

^{1}H NMR (400 MHz, $(CD_3)_2SO$): δ 13.74 (s, 1H), 7.87 (dd, J = 5.1, 1.2 Hz, 1H), 7.47 (dd, J = 3.6, 1.2 Hz, 1H), 7.25 (dd, J = 5.1, 3.6 Hz, 1H).

^{13}C NMR (100 MHz, $(CD_3)_2SO$): δ 168.48, 152.59, 134.84, 133.08, 129.71, 129.40, 127.23, 123.86.

4,7-Di(2-thienyl)-2,1,3-benzothiadiazole-5,6-dicarboxylic anhydride (4)

A solution of **3** (2.61 g, 6.72 mmol) in acetic anhydride (200 mL) was heated at reflux overnight. The reaction mixture was concentrated *in vacuo* and subsequently triturated with hexane to afford the title compound as a red solid (2.28 g, 6.16 mmol, 92% yield).

^{1}H NMR (400 MHz, $CDCl_3$): δ 8.09 (dd, J = 3.8, 1.2 Hz, 1H), 7.80 (dd, J = 5.1, 1.2 Hz, 1H), 7.31 (dd, J = 5.1, 3.8 Hz, 1H).

***N*-Butyl-4,7-di(2-thienyl)-2,1,3-benzothiadiazole-5,6-dicarboxylic imide (5a)**

A solution of **4** (199 mg, 0.538 mmol) and *n*-butylamine (0.10 mL, 1.0 mmol) in glacial acetic acid (5 mL) was heated at 100°C overnight. Acetic anhydride (2 mL) was added to the reaction mixture and heating was continued for a further 3 h. The reaction mixture was concentrated *in vacuo* and passed through a short silica plug eluting with dichloromethane. The crude product was extracted with hexane, whereupon unreacted starting material (**4**) could be isolated by filtration. The title compound was subsequently obtained by recrystallization from methanol/toluene as a yellow solid (100 mg, 0.235 mmol, 44% yield).

^{1}H NMR (400 MHz, $CDCl_3$): δ 7.90 (dd, J = 3.8, 1.2 Hz, 2H), 7.71 (dd, J = 5.1, 1.2 Hz, 2H), 7.28 (dd, J = 5.1, 3.8 Hz, 2H), 3.77 – 3.69 (m, 2H), 1.74 – 1.62 (m, 2H), 1.38 (h, J = 7.4 Hz, 2H), 0.93 (t, J = 7.4 Hz, 3H).

^{13}C NMR (100 MHz, $CDCl_3$): δ 165.90, 156.64, 133.24, 131.64, 130.35, 127.19, 127.00, 126.76, 38.79, 30.43, 20.39, 13.80.

_N_-(2-Decyltetradecyl)-4,7-di(2-thienyl)-2,1,3-benzothiadiazole-5,6-dicarboxylic imide (5b)

A solution of **4** (1.04 g, 2.80 mmol) and 2-decyltetradecylamine (1.93 g, 5.46 mmol) in glacial acetic acid (30 mL) was heated at 100°C overnight. Acetic anhydride (12 mL) was added to the reaction mixture and heating was continued for a further 3 h. The reaction mixture was concentrated *in vacuo* and extracted with hexane, whereupon unreacted starting material (**4**) could be isolated by filtration. The title compound was subsequently obtained by column chromatography (silica, hexane/dichloromethane) as an orange solid (1.44 g, 2.04 mmol, 73% yield).

^{1}H NMR (400 MHz, $CDCl_3$): δ 7.89 (dd, J = 3.7, 1.2 Hz, 2H), 7.71 (dd, J = 5.1, 1.2 Hz, 2H), 7.28 (dd, J = 5.1, 3.7 Hz, 2H), 3.62 (d, J = 7.3 Hz, 2H), 1.93 (m, 1H), 1.40 – 1.19 (m, 40H), 0.87 (m, 6H).

^{13}C NMR (100 MHz, $CDCl_3$): δ 166.14, 156.67, 133.21, 131.70, 130.33, 127.12, 127.00, 126.70, 43.39, 36.91, 32.05, 31.73, 30.15, 29.82, 29.79, 29.72, 29.49, 26.44, 22.83, 14.27.

_N_-(2-Decyltetradecyl)-4,7-di(5-bromo-2-thienyl)-2,1,3-benzothiadiazole-5,6-dicarboxylic imide (6b)

A solution of **5b** (0.96 g, 1.36 mmol) and *N*-bromosuccinimide (1.21 g, 6.80 mmol) in tetrahydrofuran (90 mL) was stirred at room temperature in the dark overnight. The reaction mixture was concentrated *in vacuo* and subsequently triturated with methanol. The title compound was obtained by column chromatography (silica, dichloromethane) as a red oil (1.15 g, 1.33 mmol, 98% yield).

^{1}H NMR (400 MHz, $CDCl_3$): δ 7.77 (d, J = 4.1 Hz, 2H), 7.22 (d, J = 4.1 Hz, 2H), 3.63 (d, J = 7.4 Hz, 2H), 1.93 (m, 1H), 1.42 – 1.12 (m, 40H), 0.87 (m, 6H).

^{13}C NMR (100 MHz, $CDCl_3$): δ 166.04, 156.14, 134.17, 133.23, 129.98, 126.47, 126.03, 118.79, 43.49, 36.95, 32.06, 31.74, 30.14, 29.82, 29.79, 29.73, 29.49, 26.45, 22.84, 14.27.

5-(1-Nonylundecyl)benzo[1,2-b:3,4-b':5,6-d'']trithiophene (C16-BTT)

Synthesized in a similar fashion to previously reported 5-(1-octylnonyl)benzo[1,2-b:3,4-b':5,6-d'']trithiophene.[2]

^{1}H NMR (400 MHz, $CDCl_3$): δ 7.71 (d, J = 5.3 Hz, 1H), 7.56 (d, J = 5.3 Hz, 1H), 7.48 (d, J = 5.3 Hz, 1H), 7.47 (d, J = 5.3 Hz, 1H), 7.42 (s, 1H), 2.99 (m, 1H), 1.73 (m, 4H), 1.24 (m, 30H), 0.86 (m, 6H).

^{13}C NMR (100 MHz, $CDCl_3$): δ 150.49, 132.71, 132.51, 131.79, 130.94, 130.88, 129.84, 124.92, 124.16, 122.95, 122.42, 119.28, 42.56, 38.19, 32.04, 29.79, 29.74, 29.68, 29.46, 27.70, 22.82, 14.26.

2,8-Dibromo-5-(1-nonylundecyl)benzo[1,2-b:3,4-b':5,6-d'']trithiophene (C16-BTT-diBr)

Synthesized in a similar fashion to previously reported 2,8-dibromo-5-(1-hexadecyl)benzo[1,2-b:3,4-b':5,6-d'']trithiophene.[3]

^{1}H NMR (400 MHz, $CDCl_3$): δ 7.65 (s, 1H), 7.49 (s, 1H), 7.28 (s, 1H), 2.96 (m, 1H), 1.70 (m, 4H), 1.24 (m, 30H), 0.85 (m, 6H).

^{13}C NMR (100 MHz, $CDCl_3$): δ 151.47, 132.13, 132.03, 131.55, 130.61, 130.22, 129.44, 125.79, 125.17, 118.89, 113.73, 112.93, 42.50, 38.06, 32.05, 29.75, 29.67, 29.47, 29.46, 27.65, 22.83, 14.26.

2,8-Bis(trimethylstannyl)-5-(1-nonylundecyl)benzo[1,2-b:3,4-b':5,6-d'']trithiophene (C16-BTT-ditin)

Synthesized in a similar fashion to previously reported 2,8-bis(trimethylstannyl)-5-(1-octylnonyl)benzo[1,2-b:3,4-b':5,6-d'']trithiophene.[4]

^{1}H NMR (400 MHz, $(CD_3)_2CO$): δ 8.01 (s, 1H), 7.75 (s, 1H), 7.72 (s, 1H), 3.07 (m, 1H), 1.76 (m, 4H), 1.28 (m, 30H), 0.83 (m, 6H), 0.49 (m, 18H).

^{13}C NMR (125 MHz, $(CD_3)_2CO$): δ 150.71, 140.69, 139.42, 136.18, 135.20, 134.70, 133.64, 133.33, 132.23, 131.12, 130.94, 121.06, 43.22, 38.97, 32.76, 28.37, 23.46, 14.51, -8.00.

BBTI-1

A degassed solution of **C16-BTT-ditin** (100.3 mg, 0.126 mmol), **5b** (108.8 mg, 0.126 mmol), $Pd_2(dba)_3$ (2.6 mg, 0.003 mmol) and $P(o\text{-}tol)_3$ (4.0 mg, 0.013 mmol) in anhydrous chlorobenzene (1.0 mL) was stirred at 180°C for 30 minutes in a microwave reactor. The crude polymer was end-capped with bromobenzene (10 μL) and trimethyl(phenyl)tin (25 μL) and subsequently precipitated into methanol. Using a Soxhlet apparatus, the crude polymer was washed with acetone and hexane and subsequently extracted with chloroform and precipitated into methanol to afford the non-fractionated polymer (146 mg, 99% yield) as a black solid. Fractionation by means of preparative size exclusion chromatography in chlorobenzene at 80°C and subsequent precipitation into methanol afforded the fractionated polymer (61 mg, 41% yield) as a black metallic solid.

GPC (chlorobenzene, 80°C): M_n = 64.3 kDa, M_w = 128 kDa, PDI = 1.99.

^{1}H NMR (400 MHz, $CDCl_3$): δ 8.0 (br s, 2H), 7.0 (br s, 5H), 3.6 (br s, 2H), 2.9 (br s, 2H), 1.3 (br m, 78H).

BBTI-2

A degassed solution of **C9C10-BTT-ditin** (99.7 mg, 0.117 mmol), **5b** (101.0 mg, 0.117 mmol), $Pd_2(dba)_3$ (2.5 mg, 0.003 mmol) and $P(o\text{-}tol)_3$ (3.3 mg, 0.011 mmol) in anhydrous chlorobenzene (1.0 mL) was stirred at 180°C for 30 minutes in a microwave reactor. The crude polymer was end-capped with bromobenzene (10 μL) and trimethyl(phenyl)tin (25 μL) and subsequently precipitated into methanol. Using a Soxhlet apparatus, the crude polymer was washed with acetone and hexane and subsequently extracted with chloroform and precipitated into methanol to afford the non-fractionated polymer (139 mg, 97% yield) as a black solid. Fractionation by means of preparative size exclusion chromatography in chlorobenzene at 80°C and subsequent precipitation into methanol afforded the fractionated polymer (49 mg, 34% yield) as a black metallic solid.

GPC (chlorobenzene, 80°C): M_n = 75.2 kDa, M_w = 159 kDa, PDI = 2.11.

^{1}H NMR (400 MHz, $CDCl_3$): δ 8.0 (br s, 2H), 7.2 (br s, 5H), 3.6 (br s, 2H), 3.1 (br s, 1H), 1.2 (br m, 87H).

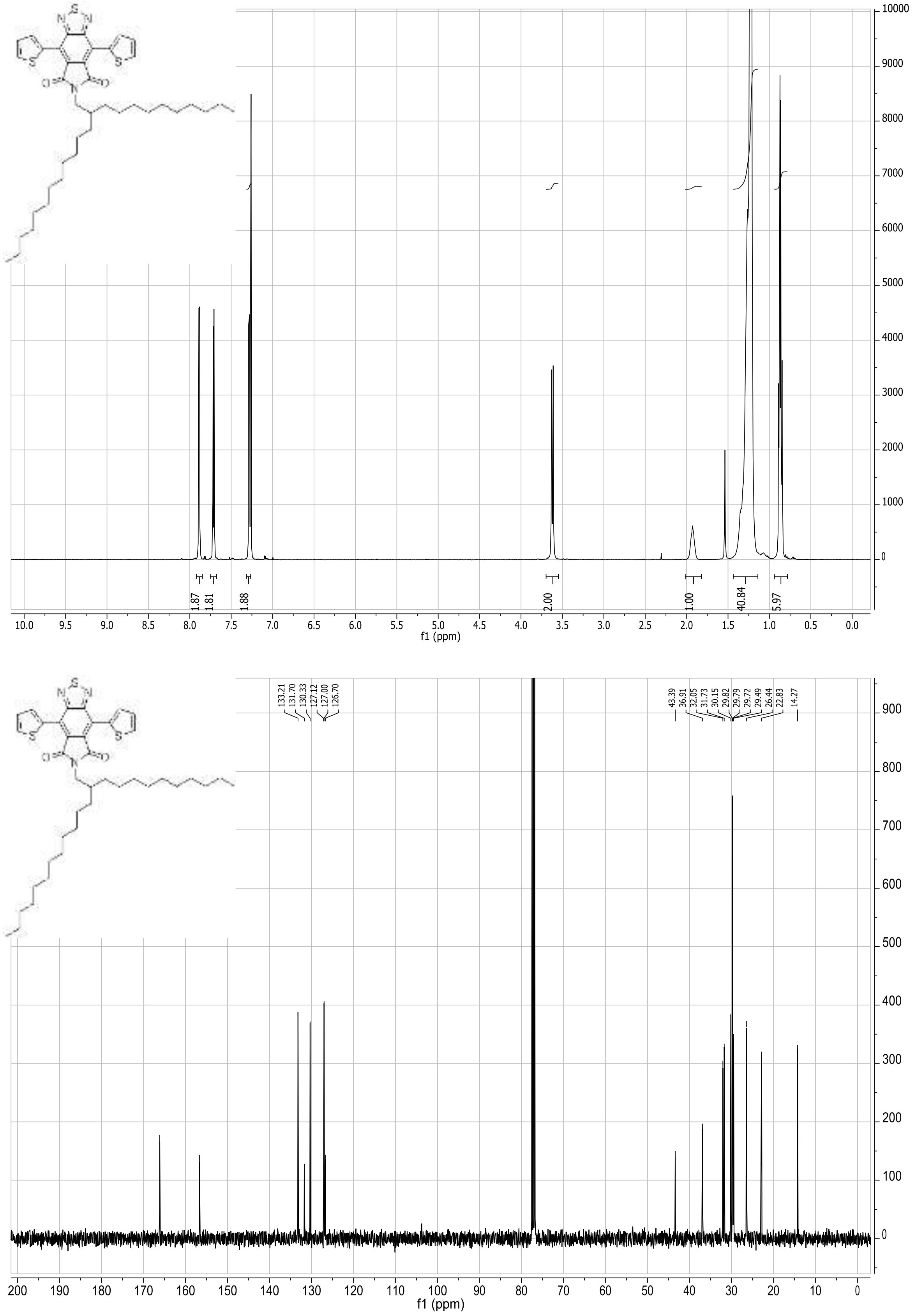

Figure S1. ^{1}H and ^{13}C NMR spectra of **5b**.

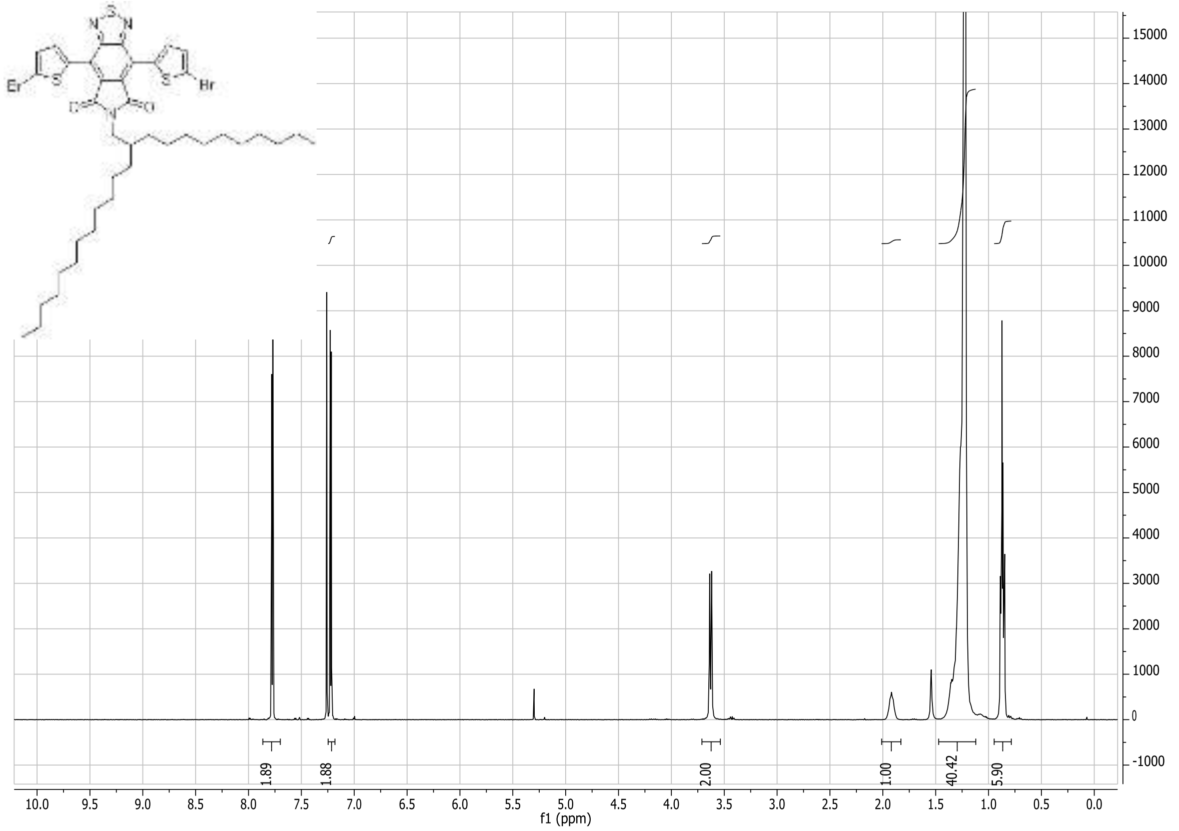

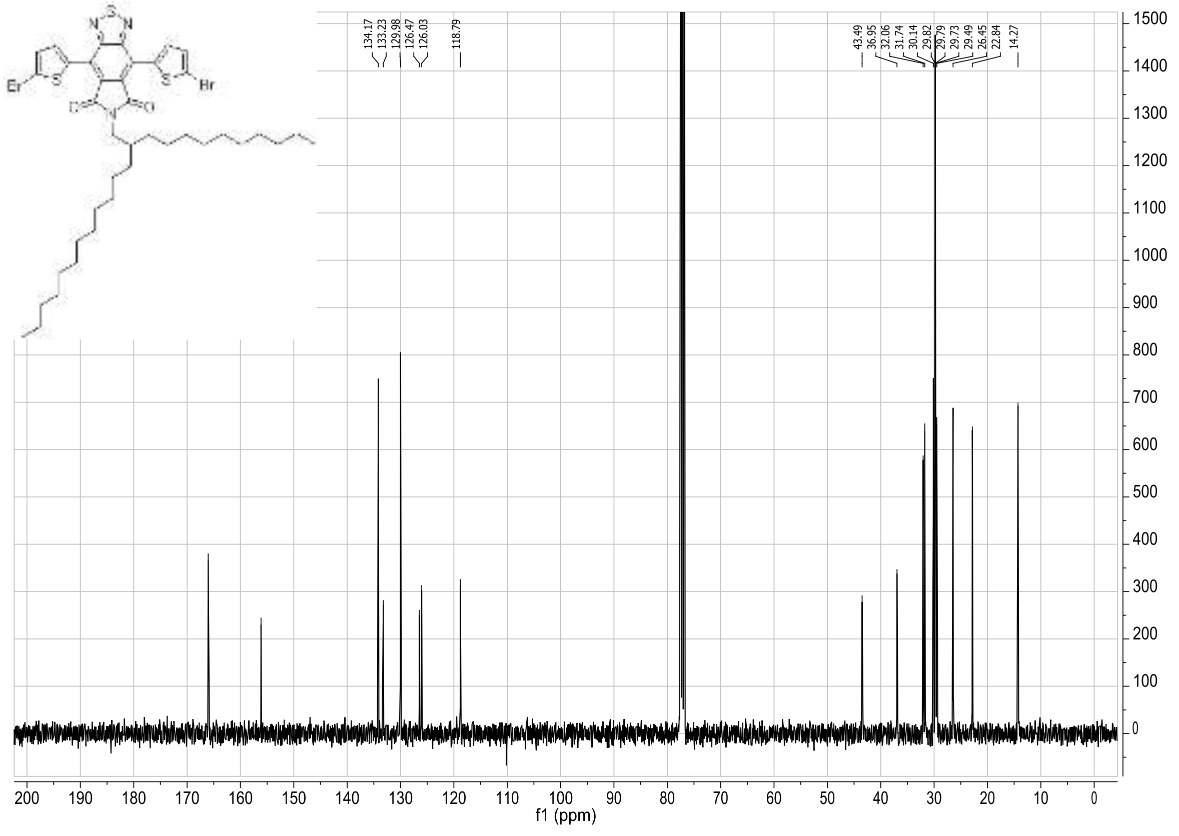

Figure S2. ^{1}H and ^{13}C NMR spectra of **6b**.

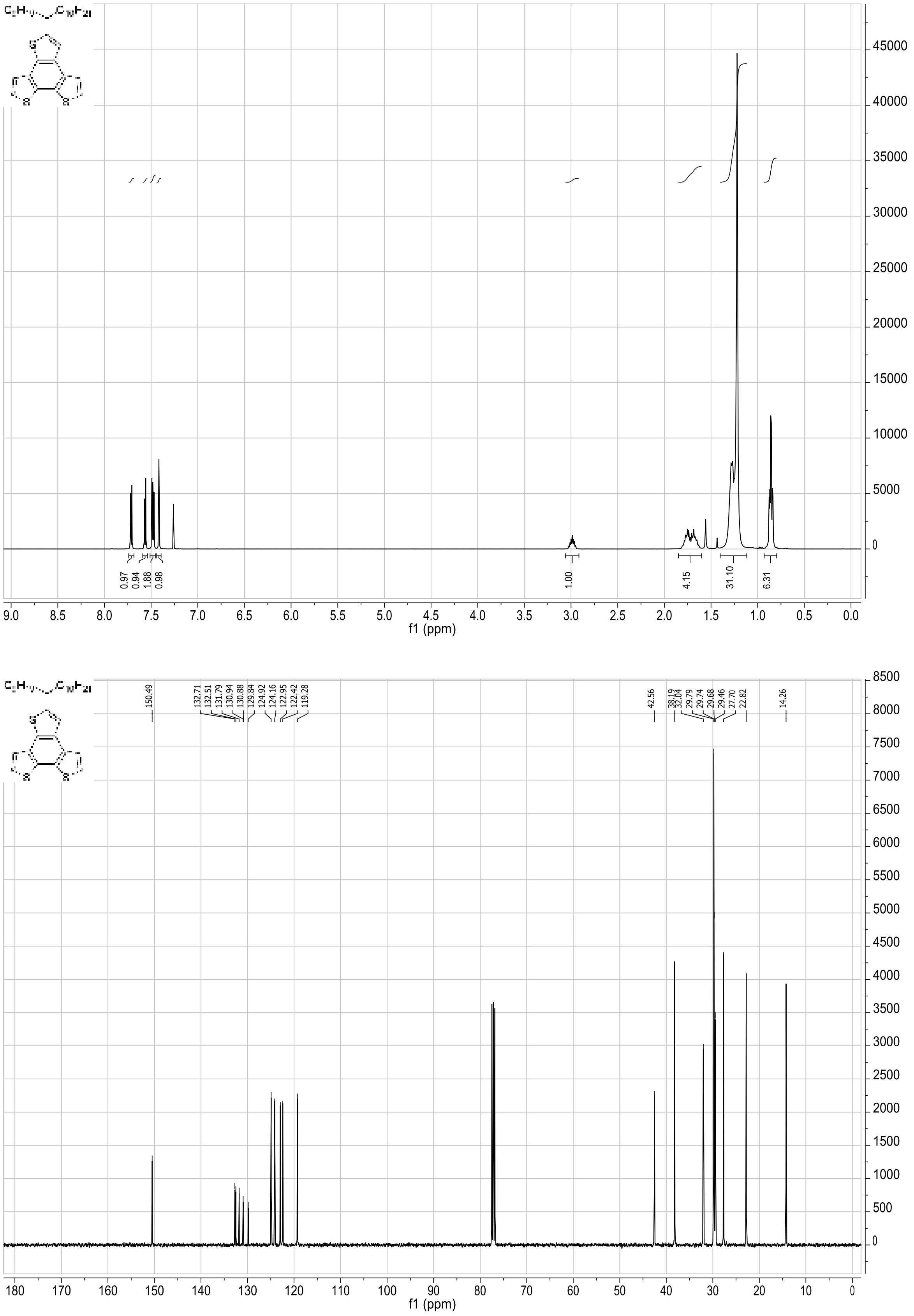

Figure S3. ^{1}H and ^{13}C NMR spectra of **C16-BTT**.

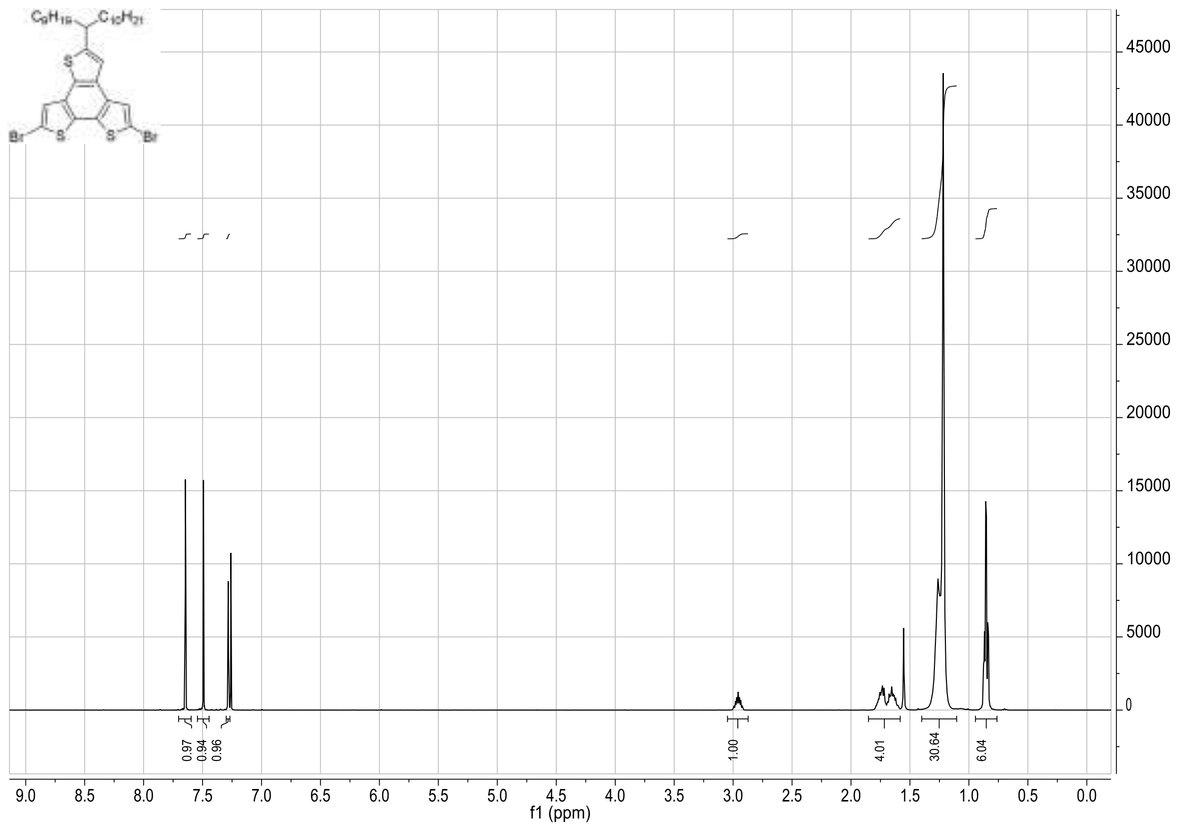

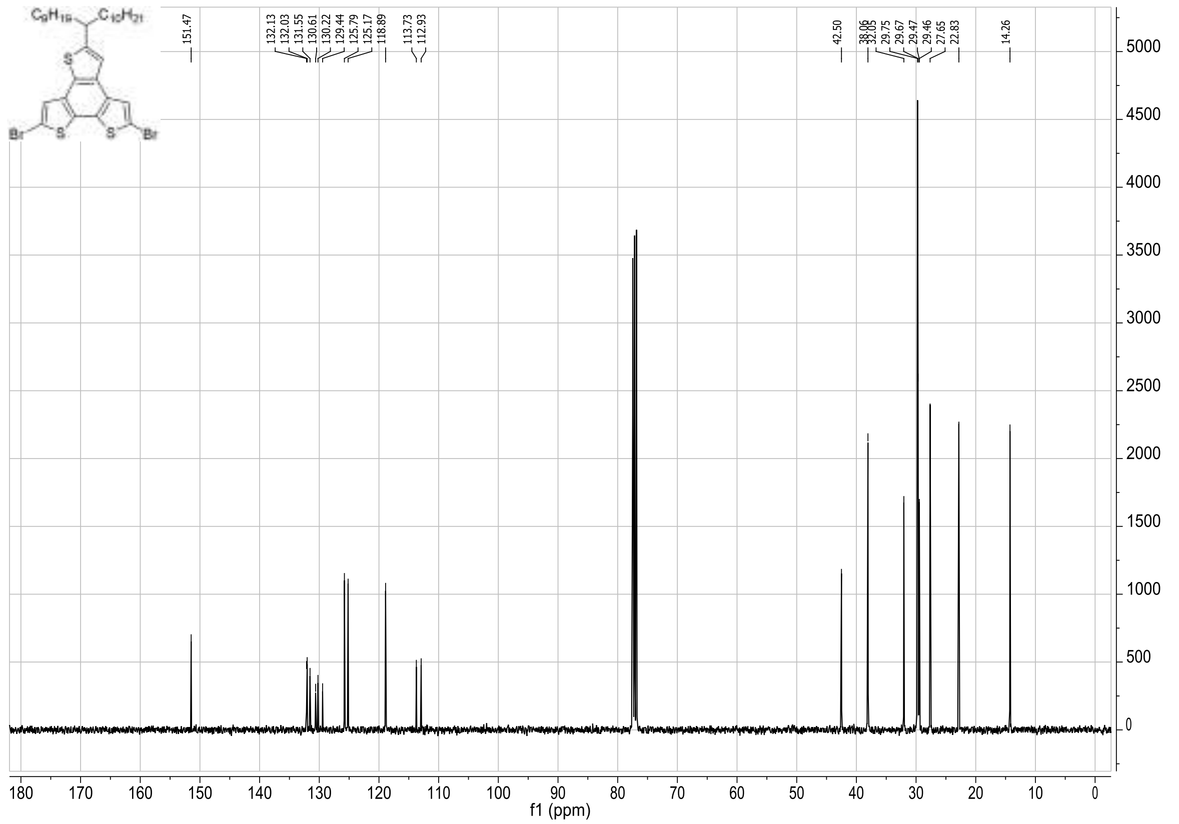

Figure S4. ^{1}H and ^{13}C NMR spectra of **C16-BTT-diBr**.

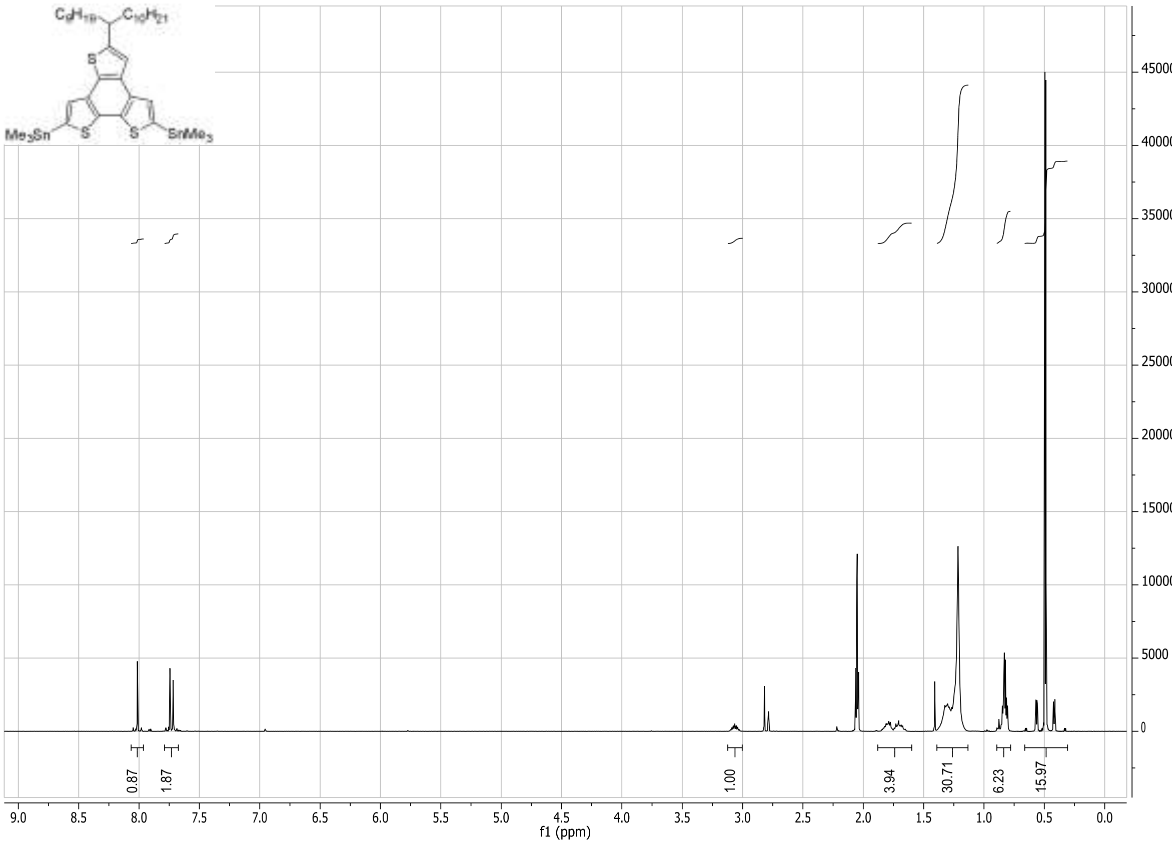

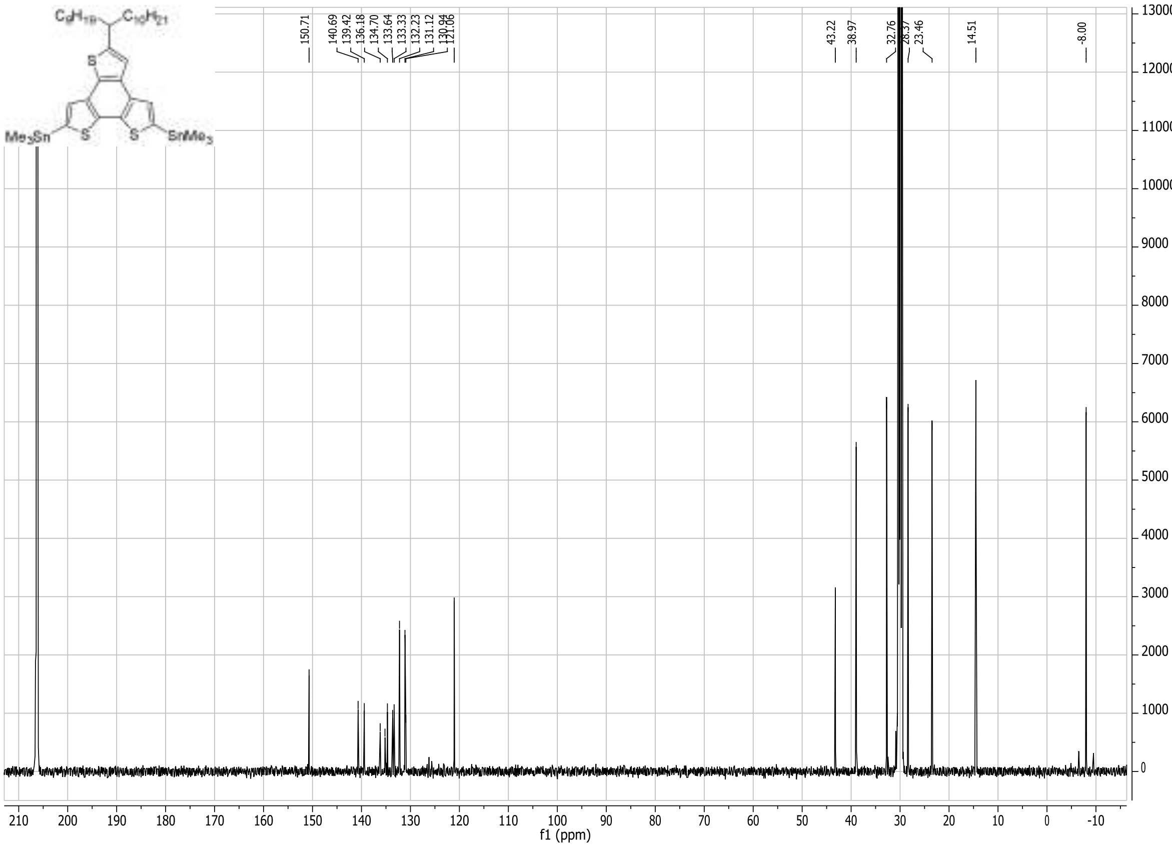

Figure S5. ^{1}H and ^{13}C NMR spectra of **C16-BTT-ditin**.

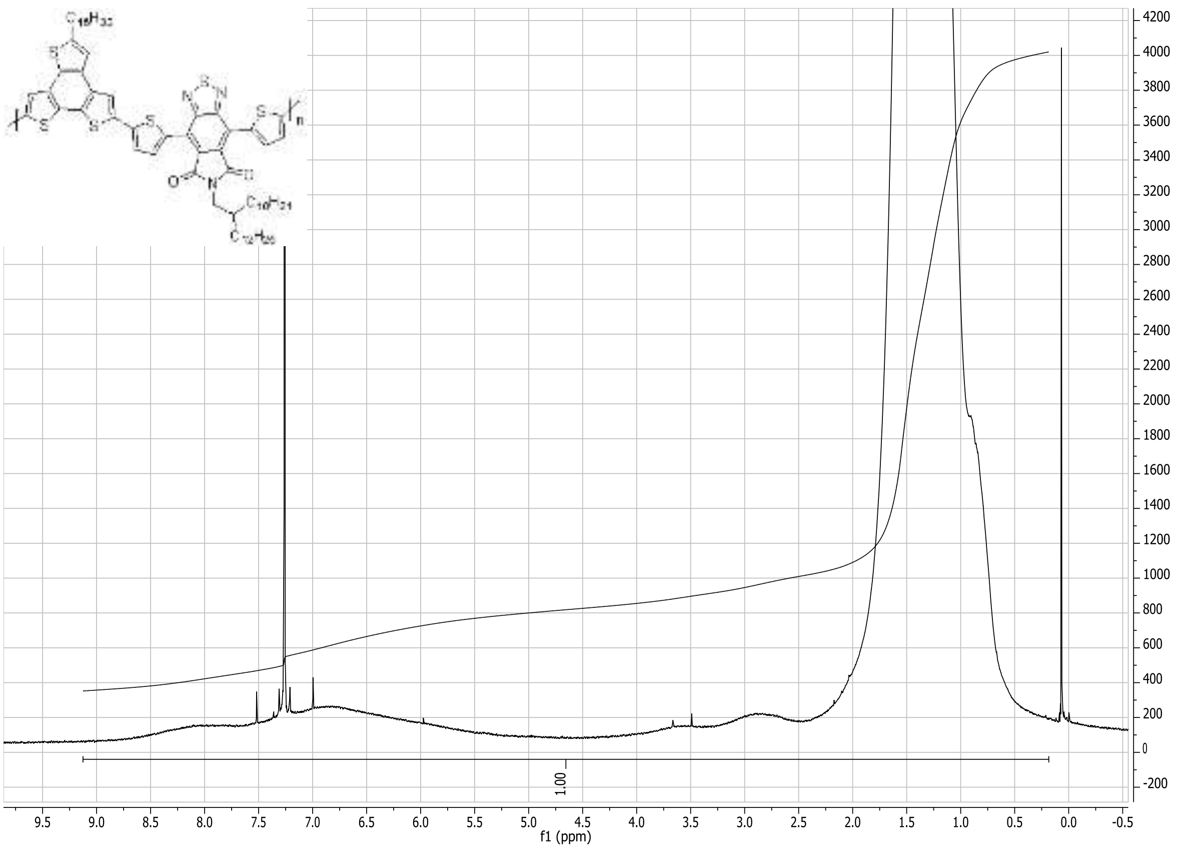

Figure S6. ^{1}H NMR spectrum of **BBTI-1**.

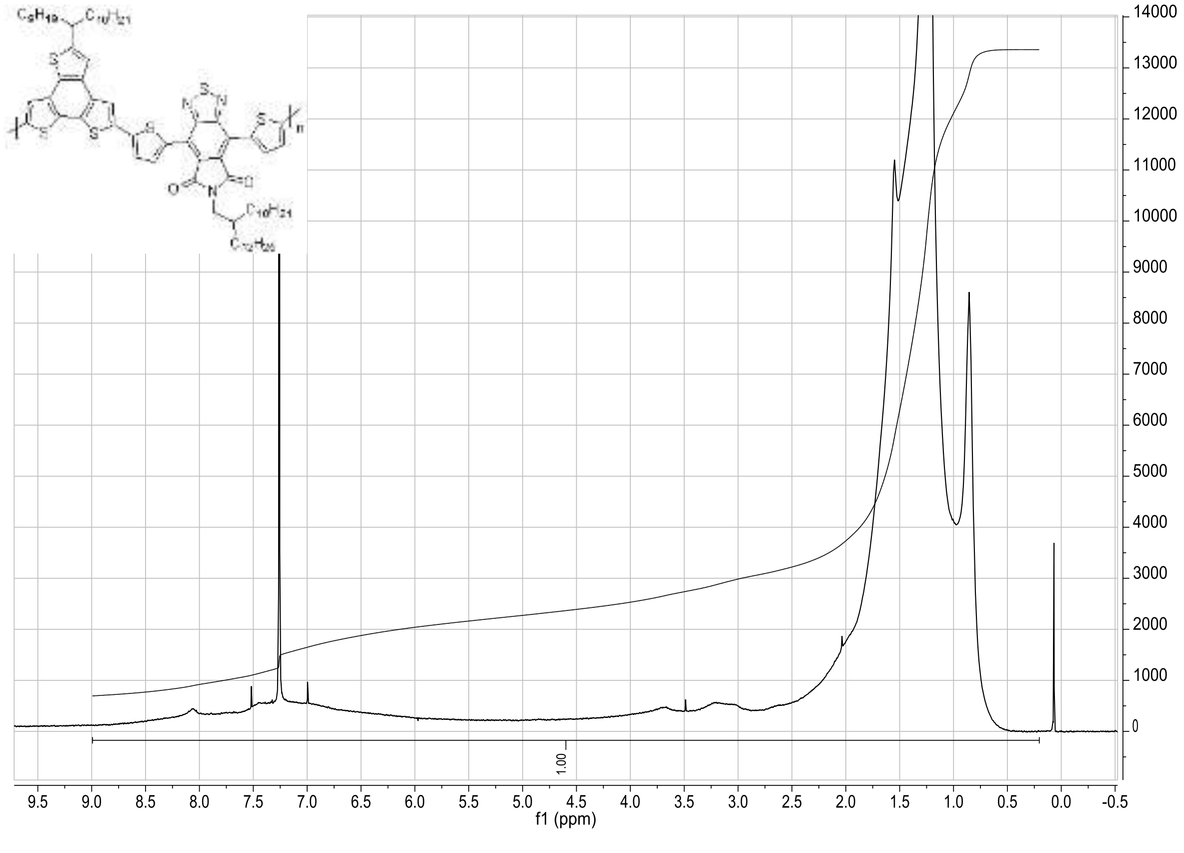

Figure S7. ^{1}H NMR spectrum of **BBTI-2**.

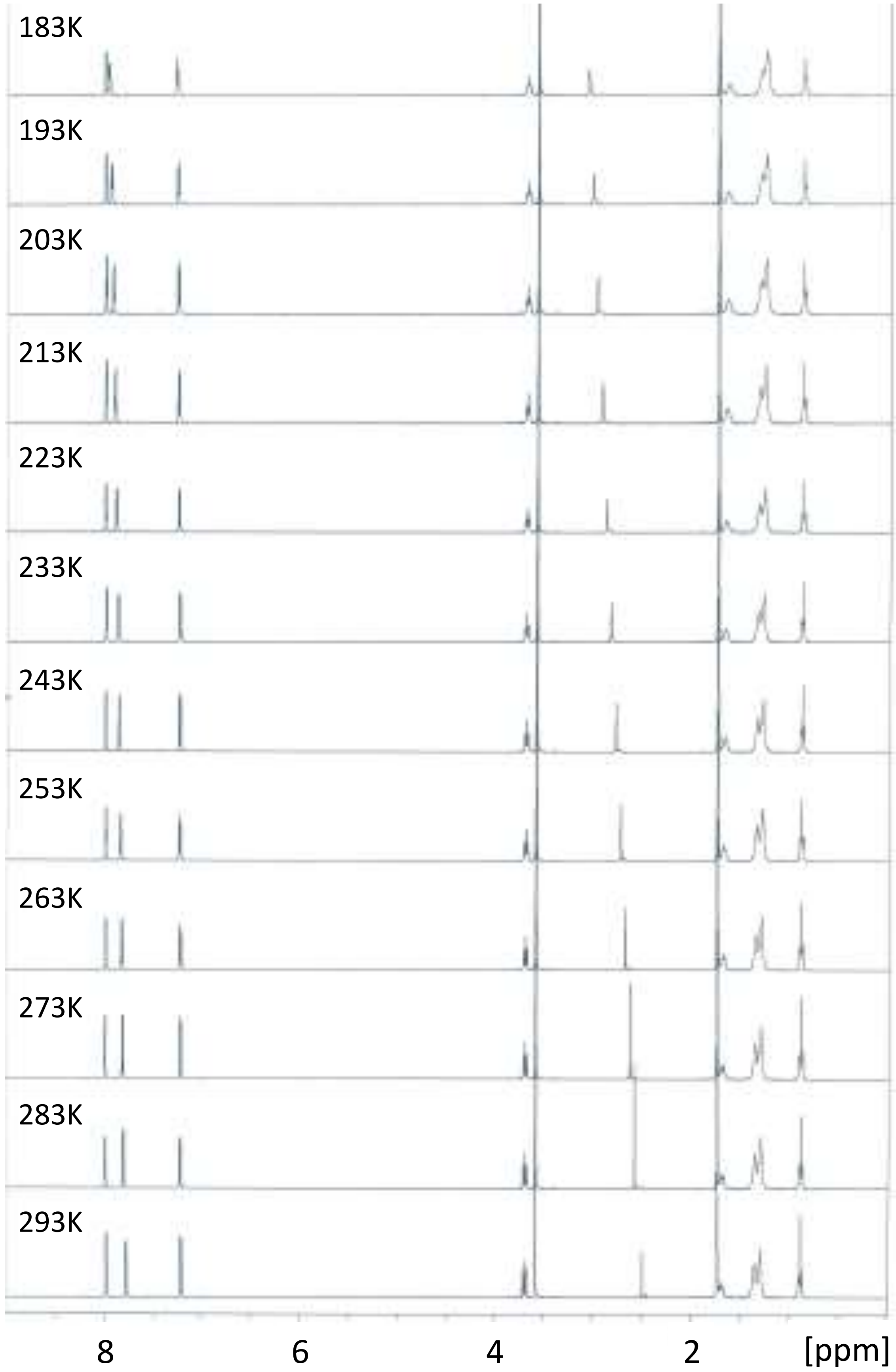

Figure S8. Variable-temperature ^{1}H NMR spectroscopy of *N*-octyl-4,7-di(2-thienyl)-2,1,3-benzothiadiazole-5,6-dicarboxylic imide in deuterated THF.

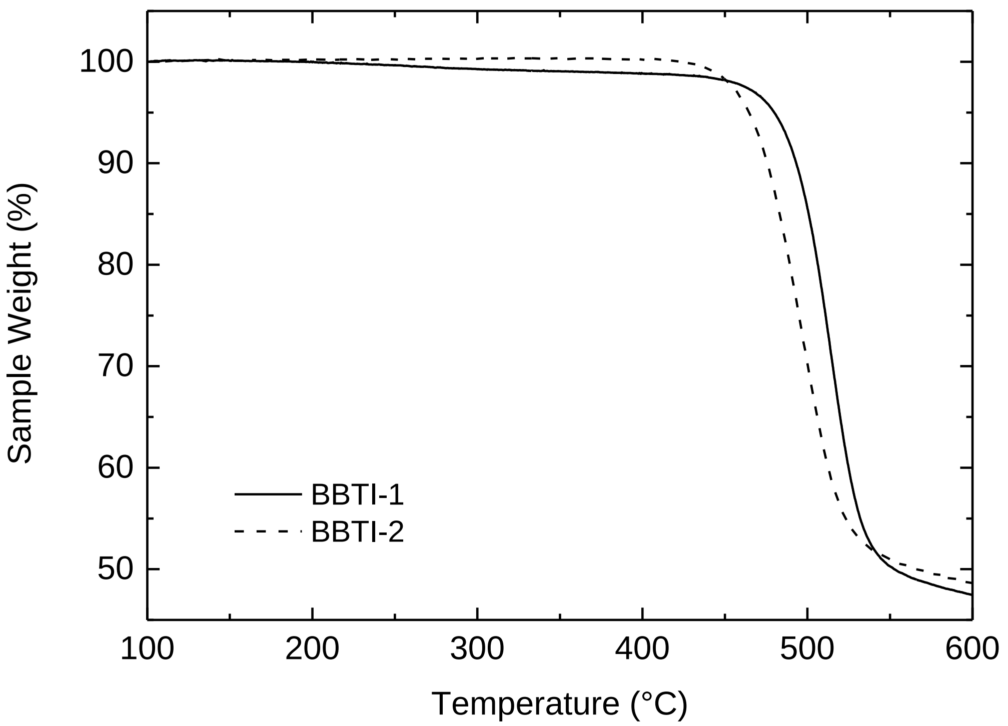

Figure S9. TGA traces of **BBTI-1** and **BBTI-2** recorded at 10°C/min in a nitrogen atmosphere.

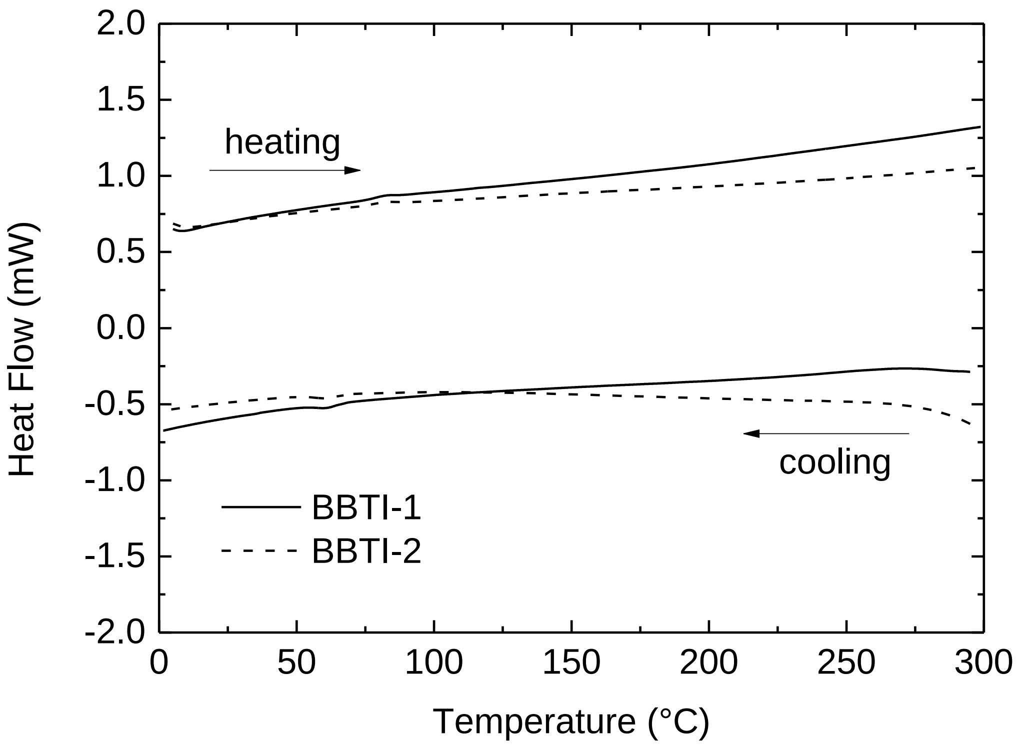

Figure S10. DSC traces (with exotherm down) of **BBTI-1** and **BBTI-2** recorded at 10°C/min; small peak around 80°C (heating) and 60°C (cooling) is an instrument artefact.

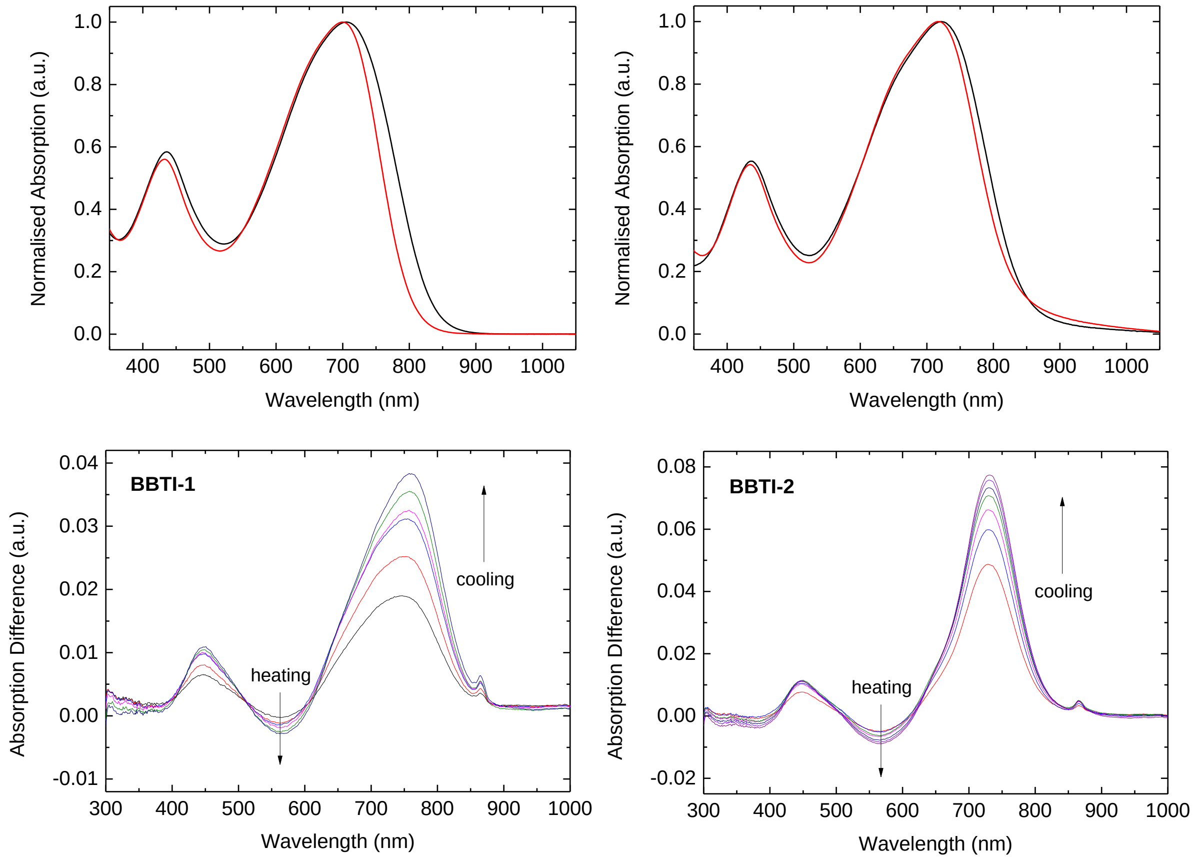

Figure S11. UV-vis spectra of **BBTI-1** (black) and **BBTI-2** (red) recorded in chlorobenzene solution (top left) and as thin films spin-cast from chlorobenzene (top right); bottom pane depicts the temperature-dependant solution spectra for **BTTBTI-1** (left) and **BTTBTI-2** (right).

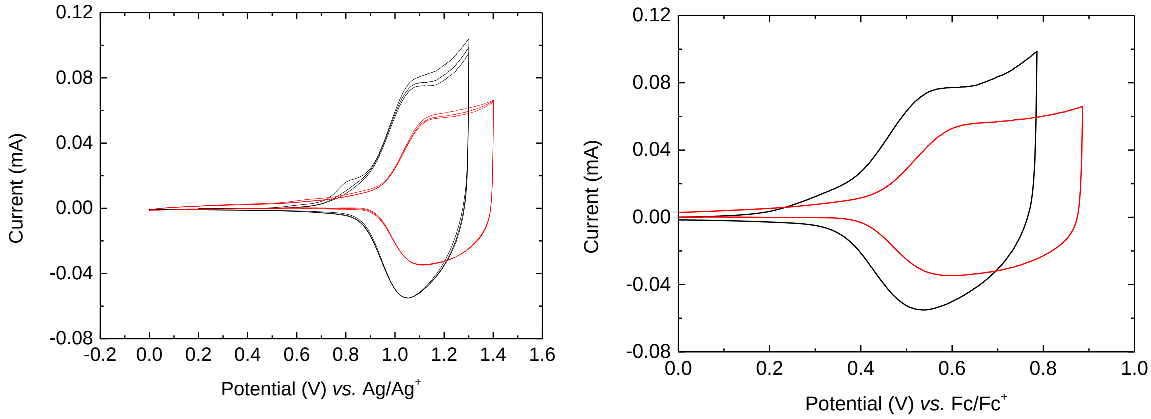

Figure S12. Cyclic voltammograms of **BBTI-1** (black lines) and **BBTI-2** (red lines) thin films recorded at 50 mV/s with 0.1M tetrabutylammonium hexafluorophosphate in acetonitrile as supporting electrolyte; left figure shows three consecutive scans for each polymer while right figure only shows the second scan for each polymer.

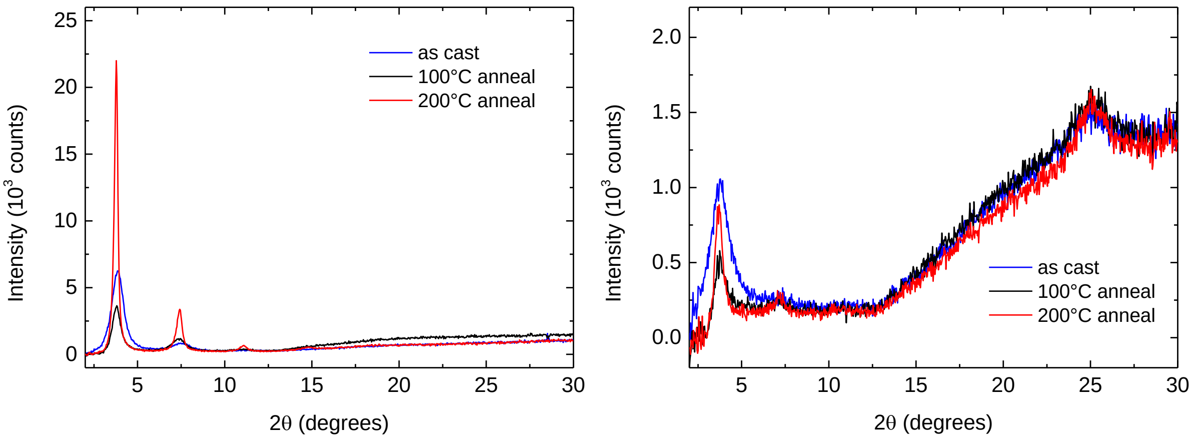

Figure S13. XRD diffractograms of thick drop cast films (from 5 mg/ml chlorobenzene solution) of **BBTI-1** (left) and **BBTI-2** (right) measured on the as cast films and after annealing at 100°C and 200°C for 20 minutes in an inert atmosphere.

Table S1. Photovoltaic Device Parameters for Conventional Configuration Solar Cells with **BTTBTI-1** ad **BTTBTI-2**.[a]

	Additive	J_{sc} (mA/cm^2)	V_{oc} (V)	FF	PCE (%)
**BTTBTI-1**	none	10.92	0.80	0.59	5.15
**BTTBTI-1**	3% DIO	5.14	0.73	0.39	1.47
**BTTBTI-2**	none	7.24	0.88	0.57	3.63
**BTTBTI-2**	3% DIO	9.72	0.81	0.65	5.12

[a]Device configuration: ITO/PEDOT:PSS/Polymer:$PC_{71}BM$(1:2)/Ca/Al.

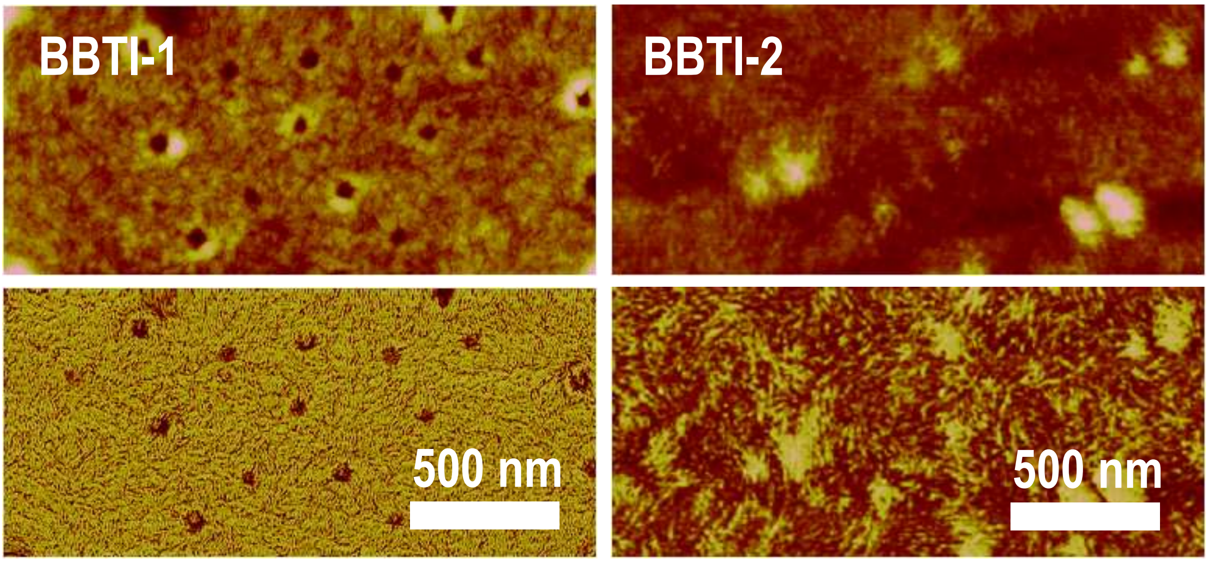

Figure S14. AFM micrographs of neat spin cast films (from 5 mg/ml chlorobenzene solution) of **BBTI-1** (left) and **BBTI-2** (right) showing topography (top) and phase (bottom).

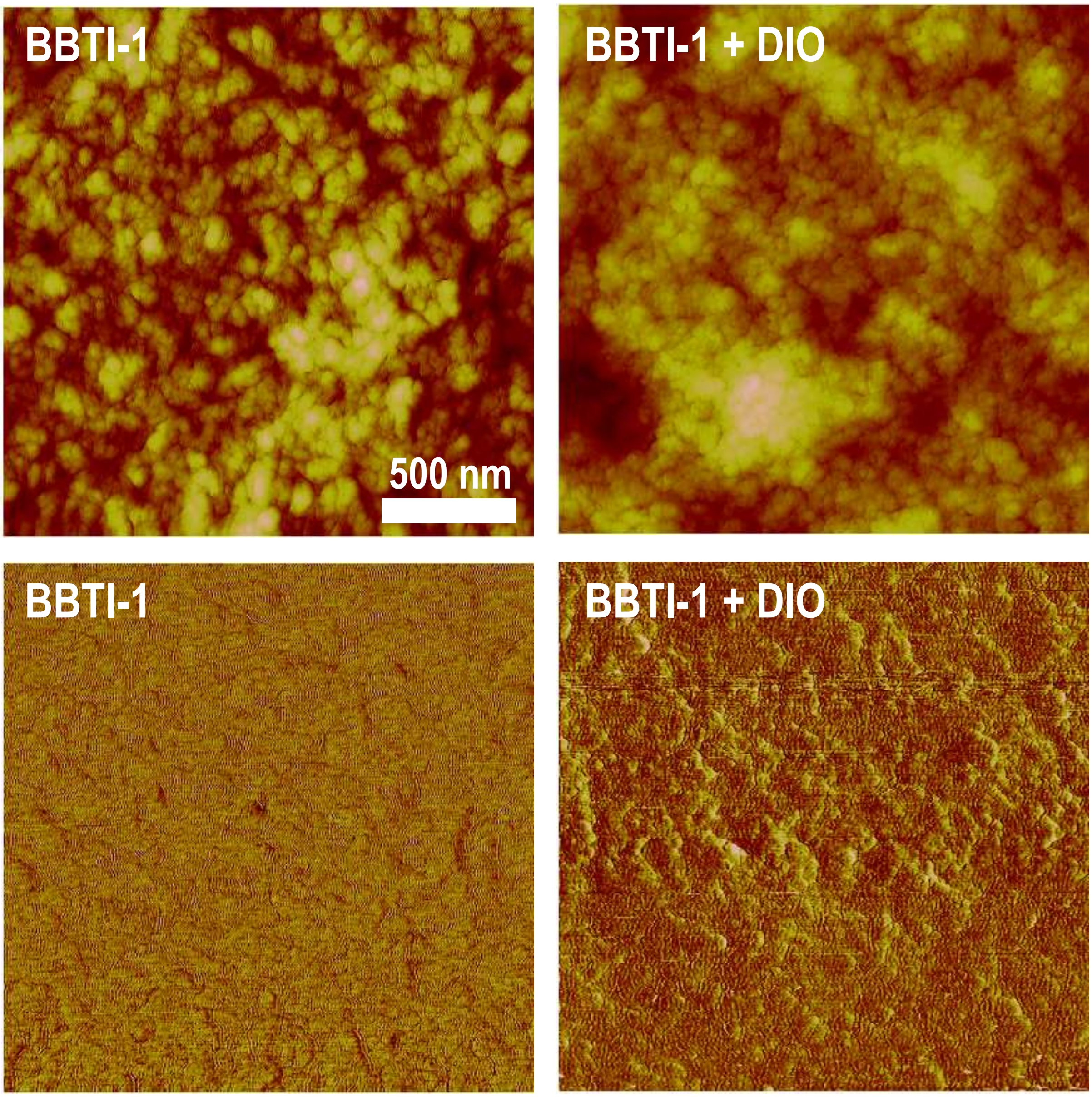

Figure S15. AFM micrographs (2 µm × 2 µm) of inverted devices with **BBTI-1** without (left) and with (right) 3% DIO solvent additive showing topography (top) and phase (bottom); RMS values are 1.67 nm (without) and 2.01 nm (with DIO additive) respectively.

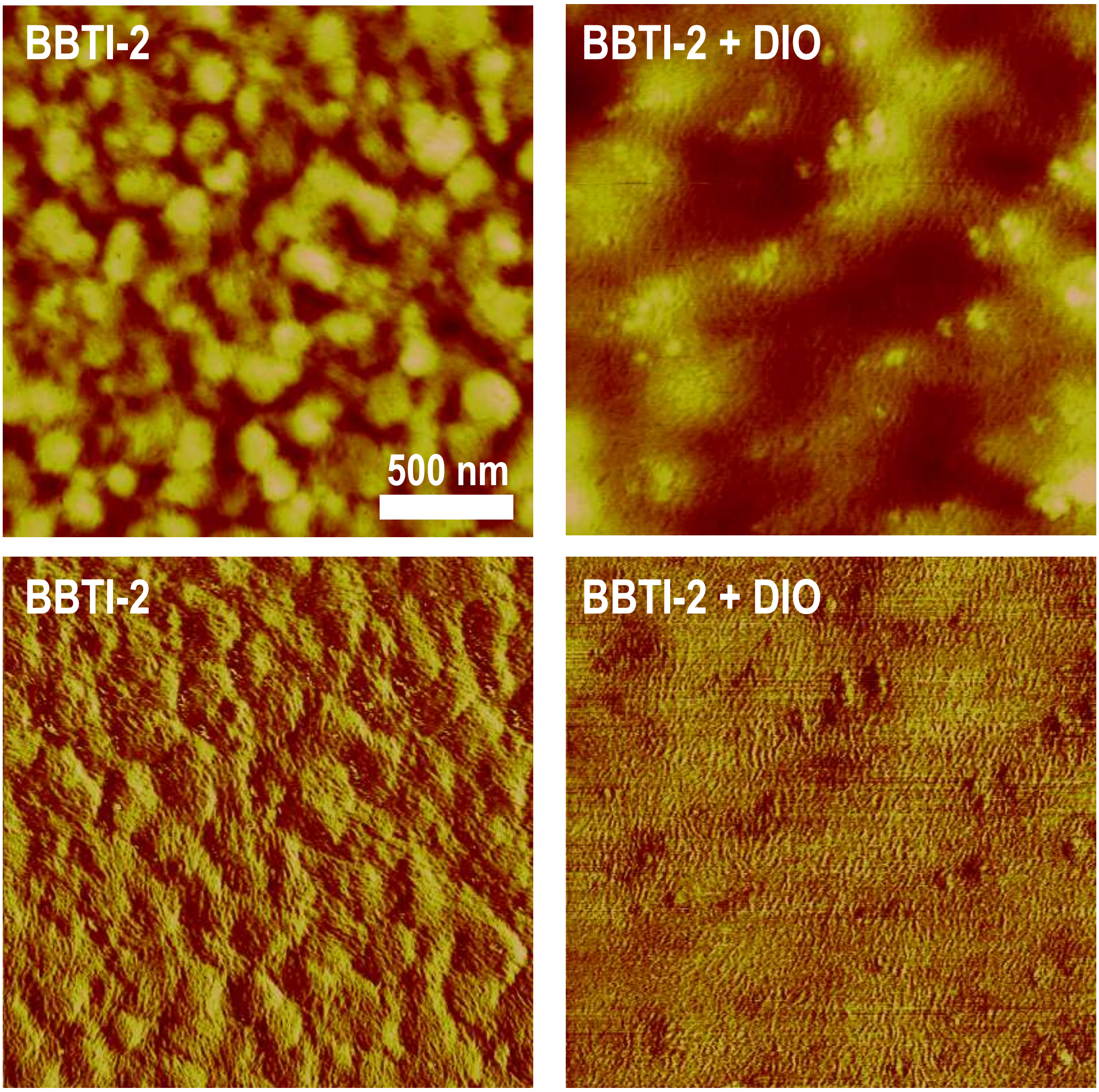

Figure S16. AFM micrographs (2 μm × 2 μm) of inverted devices with **BBTI-2** without (left) and with (right) 3% DIO solvent additive showing topography (top) and phase (bottom); RMS values are 3.09 nm (without) and 1.37 nm (with DIO additive) respectively.

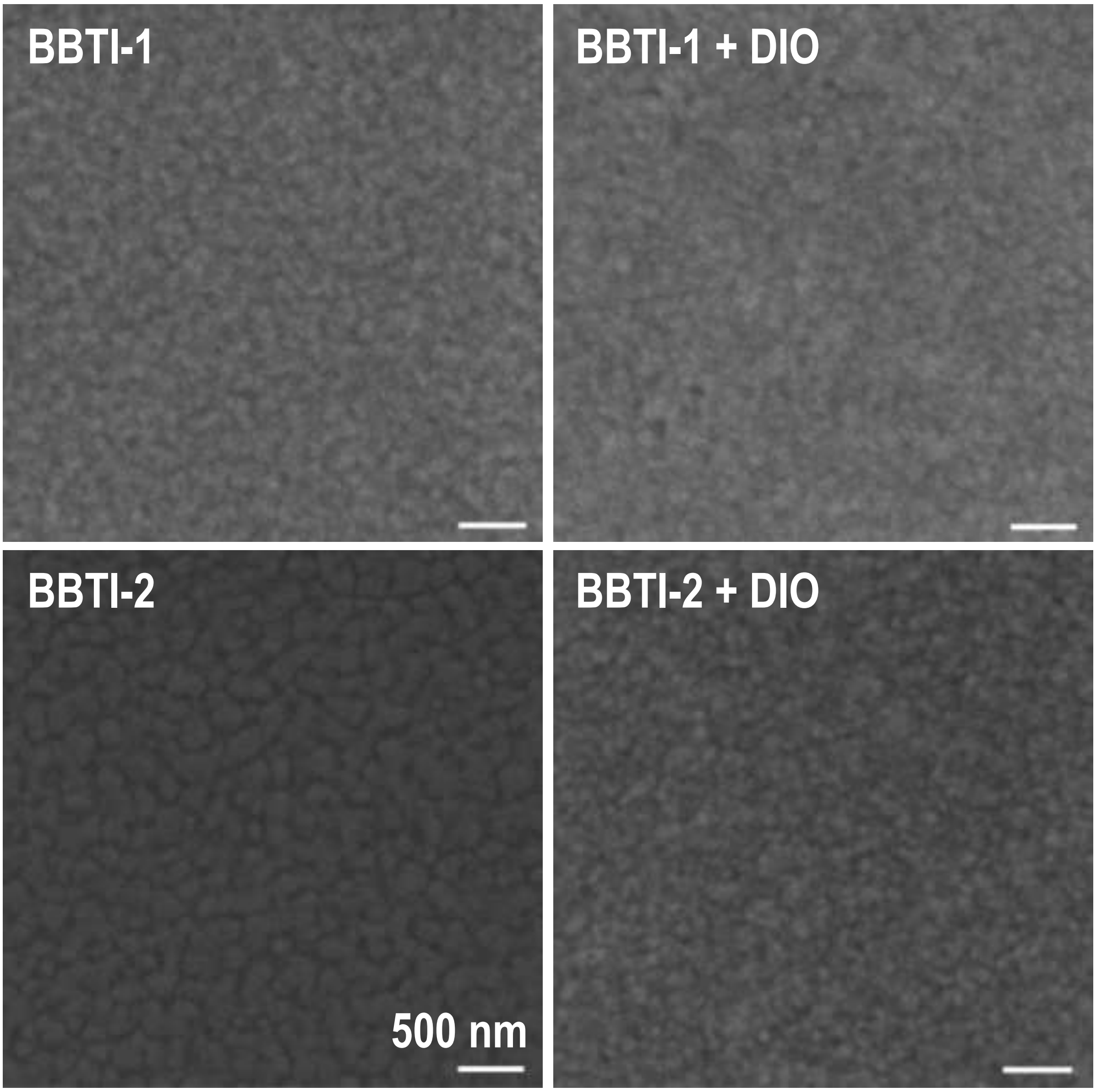

Figure S17. High resolution scanning electron micrographs (45k magnification) showing the surface morphology of bulk heterojunction blend films (polymer:$PC_{71}BM$ in a 1:2 weight ratio cast from *o*-dichlorobenzene solution) for **BBTI-1** (top) and **BBTI-2** (bottom) without solvent additive (left) and with 3% 1,8-diiodooctane additive (right).

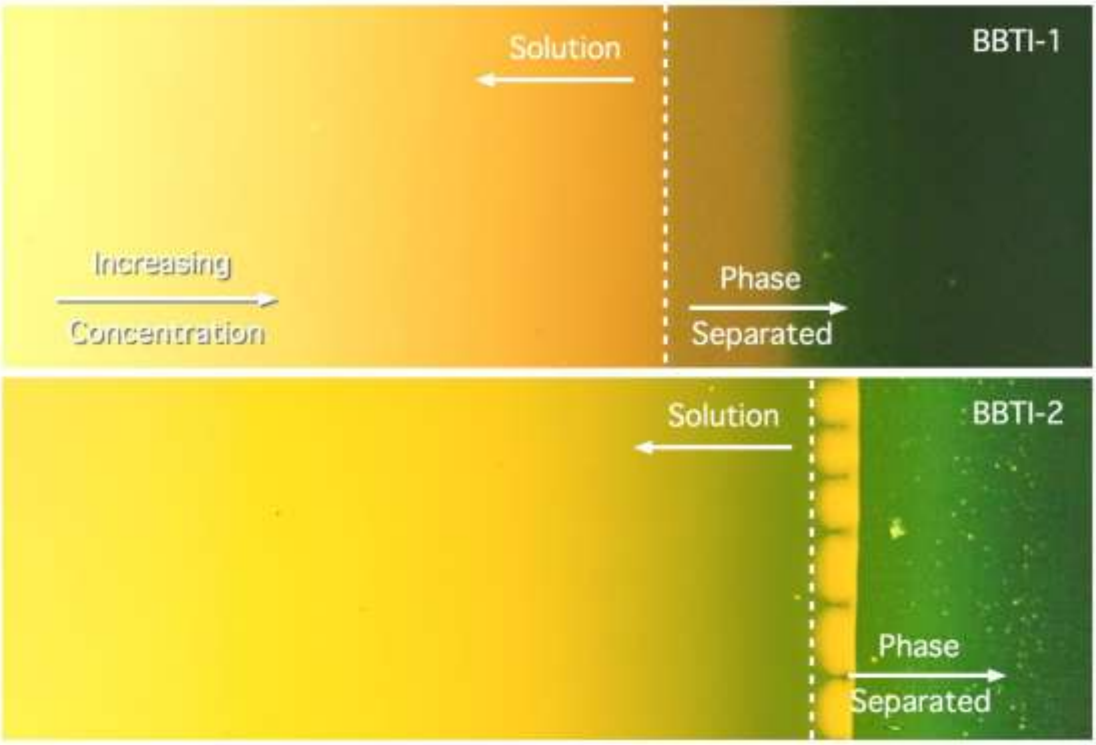

Figure S18. Transmission optical micrograph of the (top) **BBTI-1**:$PC_{71}BM$ (1:2) blend and (bottom) **BBTI-2**:$PC_{71}BM$ (1:2) in ODCB during the evaporation of ODCB. The polymer:fullerene concentration increases from 30 mg • mL^{-1} (left) to a dry film (right).

To determine the influences of the side-chain architecture on the microstructure and power conversion efficiency, focus was turned to the driving force towards phase separation that controls the final microstructure during solution casting. For this, an experiment was designed to directly observe the concentration at which phase separation occurs during solvent evaporation within the ternary polymer:fullerene:solvent system. A homogenous solution (same concentration used for devices) was placed in a confined cavity with a single liquid-air interface, allowing solvent to evaporate in one direction. Importantly, this sample geometry allows direct observation of phase separation of the polymer and fullerene during solvent evaporation in a single image, effectively simulating the concentration of solvent at which either solid-liquid or liquid-liquid phase separation occurs.

The transmission optical micrographs from blends comprising **BBTI-1** and **BBTI-2** are presented in Figure S18. As the solvent evaporates, it was observed that there is a concentration above which the system is no longer a homogeneous solution, i.e. the system is phase-separated. Importantly, these images reveal that, for blends of **BBTI-1**, phase separation occurs at a lower concentration of solids (i.e. earlier during the spin coating process) relative to the **BBTI-2** indicating a larger thermodynamic driving force for phase separation. This observation is also supported by temperature-dependent optical micrographs that reveal crystallization of **BBTI-1** occurs at experimentally observable temperatures while no such phase separation is observed in the **BBTI-2** blends (see Figure S19). Combining our observations strongly suggests that the lesser space-filling linear C16 chain (**BBTI-1**) increases the thermodynamic driving force for polymer:fullerene blends to crystallize resulting in a more optimum microstructure for efficient power conversion.

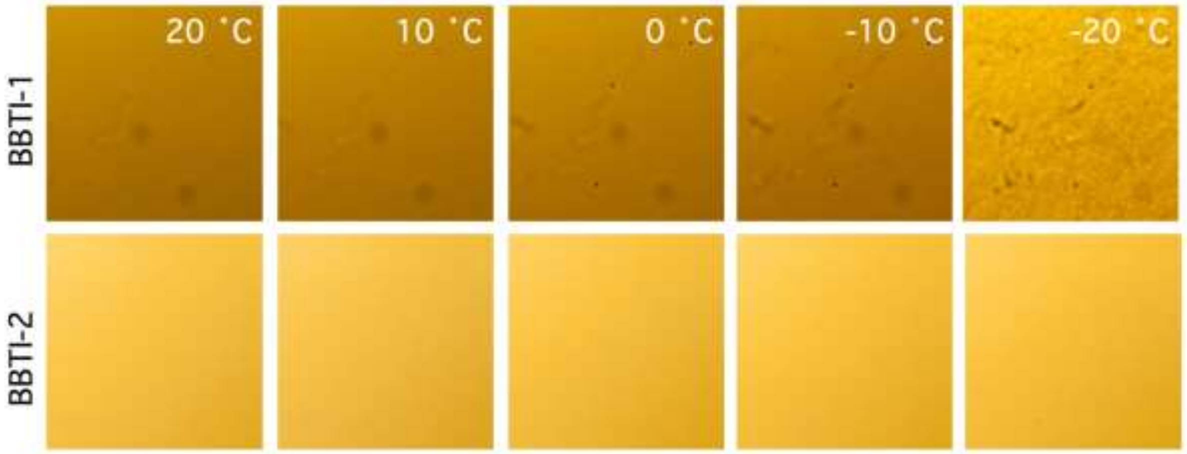

Figure S19. Transmission optical micrograph images of 30 mg • mL^{-1} (top) **BBTI-1**:$PC_{71}BM$ (1:2) and (bottom) **BBTI-2**:$PC_{71}BM$ (1:2) in ODCB collected as a function of temperature.

Following the thermodynamic understanding of mixing, at a given ternary composition, a temperature should be reached at which all compositions of the individual components are no longer miscible; the result of this will either be liquid-liquid or solid-liquid phase separation. Therefore, we set out to probe whether, at various the temperatures, we will observe liquid-liquid phase separation or crystallization (*i.e.* solid- liquid phase separation) of the ternary solution at constant concentration.

We used temperature-dependent optical microscopy at a constant total polymer:fullerene concentration of 30 mg • mL^{-1} (1:2 blend) in ODCB. The solution was deposited in a cavity and sealed to limit the evaporation of ODCB. The samples were then heated to 35 ˚C in a temperature-controlled microscope stage to form a homogeneous solution and subsequently cooled to -20 ˚C at a rate of -0.5 ˚C/min; transmission optical micrographs were taken of this process at a 10 ˚C interval (*i.e.* at every 20 min). From these images (Figure S19), we observed solid-liquid phase separation in the **BBTI-1**:$PC_{71}BM$ solution at -20 ˚C where the **BBTI-2**:$PC_{71}BM$ solution remains homogenous. From the birefringence and microstructure observed in the optical microscope images, it is evident that the phase separation observed is due to the crystallization of the **BBTI-1**. Thus, these data indicate that the blends of **BBTI-1** and $PC_{71}BM$ have a larger thermodynamic driving force for solid-liquid phase separation relative to **BBTI-2**.

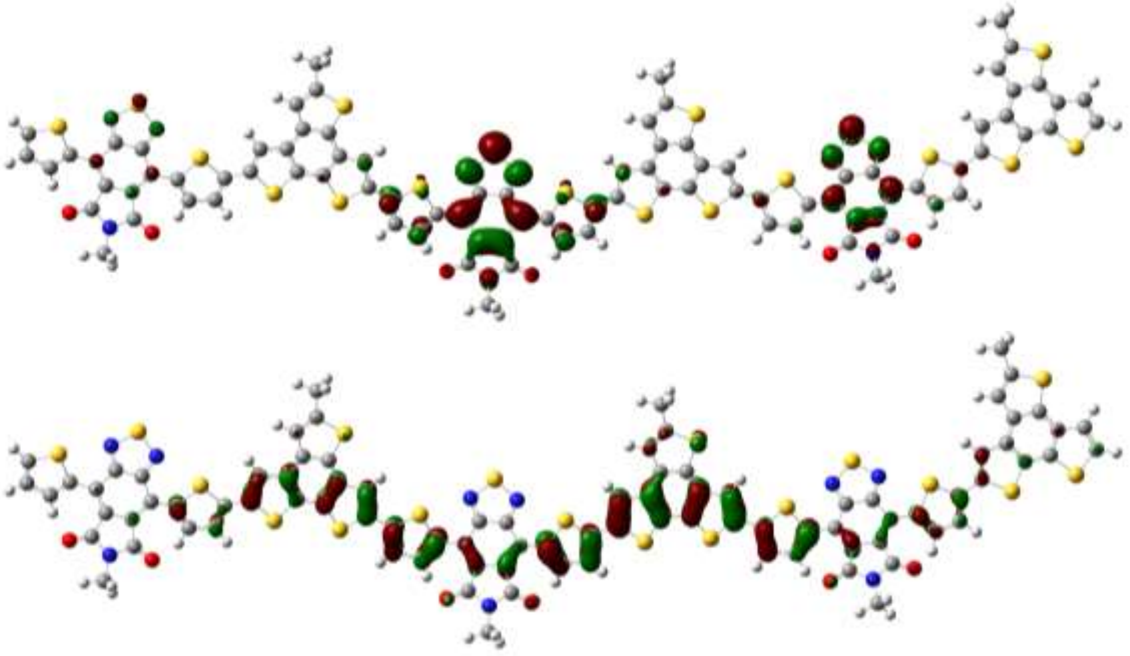

Figure S20. LUMO (top) and HOMO (bottom) distributions for the minimum-energy conformation of a trimer of **BBTI** with methyl side-chains optimized with Gaussian at the B3LYP/6-31G* level of theory and visualized with an isovalue of 0.025.

The X-ray crystal structure of dimethyl 4,7-di(2-thienyl)-2,1,3-benzothiadiazole-5,6-dicarboxylate (2)

Crystal data for **2**: $C_{18}H_{12}N_2O_4S_3$, M = 416.48, monoclinic, $P2_1/n$ (no. 14), a = 11.2235(4), b = 9.7883(4), c = 17.0603(7) Å, β = 102.480(3)°, V = 1829.95(12) Å^3, Z = 4, D_c = 1.512 g cm^{-3}, μ(Mo-Kα) = 0.433 mm^{-1}, T = 173 K, orange blocky needles, Agilent Xcalibur 3E diffractometer; 4064 independent measured reflections (R_{int} = 0.0262), F^2 refinement,[5] R_1(obs) = 0.0482, wR_2(all) = 0.1151, 3236 independent observed absorption-corrected reflections [$|F_o| > 4\sigma(|F_o|)$, $2\theta_{max}$ = 57°], 289 parameters. CCDC 1017114.

Both the C(10)- and C(21)-based thiophene rings in the structure of **2** were found to be disordered. In each case two orientations were identified, of *ca*. 61:39 and 73:27% occupancy for the C(10)- and C(21)-based rings respectively. The geometries of all four rings were optimized, the thermal parameters of adjacent atoms were restrained to be similar, and only the non-hydrogen atoms of the major occupancy orientations were refined anisotropically (those of the minor occupancy orientations were refined isotropically). In each case, the disorder amounts to a swapping of the sulfur position between the two α sites.

The X-ray crystal structure of *N*-butyl-4,7-di(2-thienyl)-2,1,3-benzothiadiazole-5,6-dicarboxylic imide (5a)

Crystal data for **5a**: $C_{20}H_{15}N_3O_2S_3$, M = 425.53, monoclinic, $P2_1/c$ (no. 14), a = 10.7065(5), b = 44.225(3), c = 7.8180(4) Å, β = 91.641(4)°, V = 3700.3(3) Å^3, Z = 8 (two independent molecules), D_c = 1.528 g cm^{-3}, μ(Cu-Kα) = 3.858 mm^{-1}, T = 173 K, orange plates, Agilent Xcalibur PX Ultra A diffractometer; 7328 independent measured reflections (R_{int} = 0.0699), F^2 refinement,[5] R_1(obs) = 0.1226, wR_2(all) = 0.2725, 5890 independent observed absorption-corrected reflections [$|F_o| > 4\sigma(|F_o|)$, $2\theta_{max}$ = 148°], 505 parameters. CCDC 1017115.

Right from the early diffraction images it was clear that the crystal used for the X-ray diffraction analysis of compound **5a** was twinned, with broad streaky “spots” with multiple humps. However, the nature of the sample was such that it was highly unlikely that anything better would be found, and so this sub-optimal crystal was used for the data collection (which lasted for *ca*. 70 hours). Reciprocal space analysis of the full data set showed multiple overlapping lattices. Numerous attempts to model this twinning at the data processing stage did not provide any beneficial results, so the data was processed based only on the major orientation.

The structure of **5a** was found to contain two crystallographically independent molecules, (**5a-A** and **5a-B**) shown in Figures S22 and S23 respectively. These molecules have very similar conformations, the best fit of all of the non-hydrogen atoms of the two molecules having an r.m.s. deviation of only *ca*. 0.04 Å.

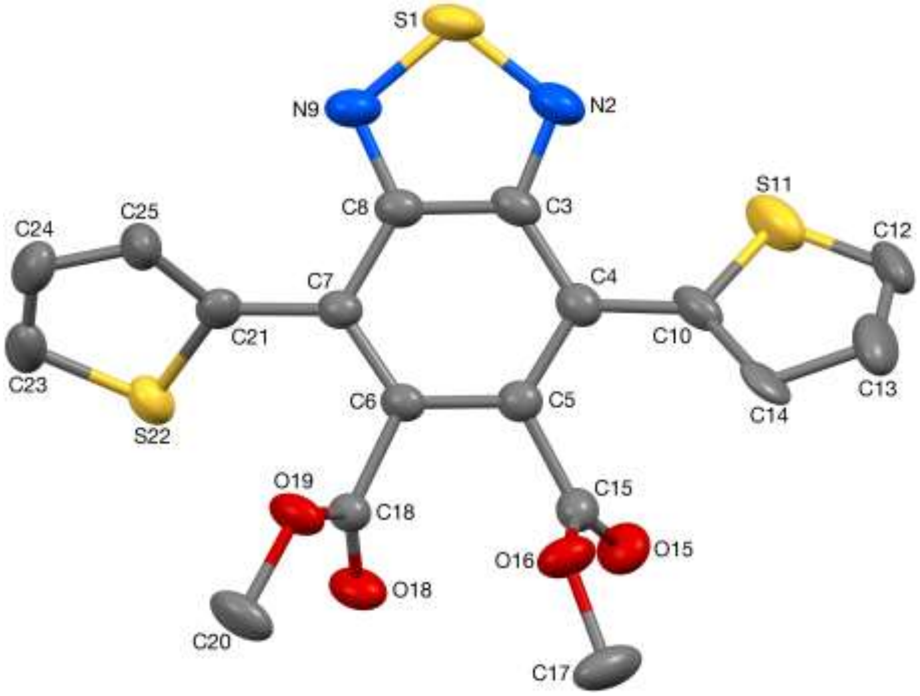

Figure S21. The crystal structure of **2** (50% probability ellipsoids).

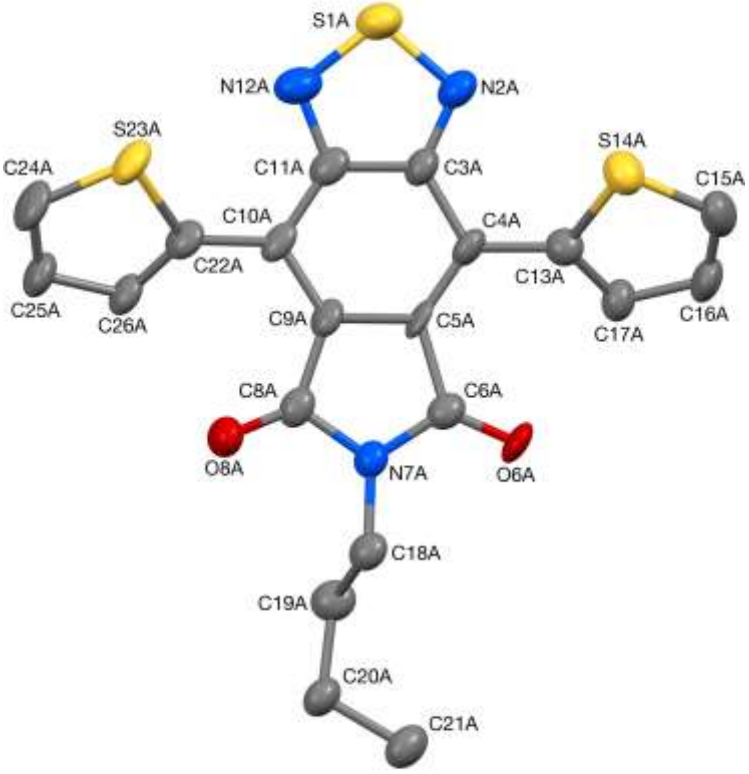

Figure S22. The structure of one (**5a-A**) of the two independent molecules present in the crystal of **5a** (50% probability ellipsoids).

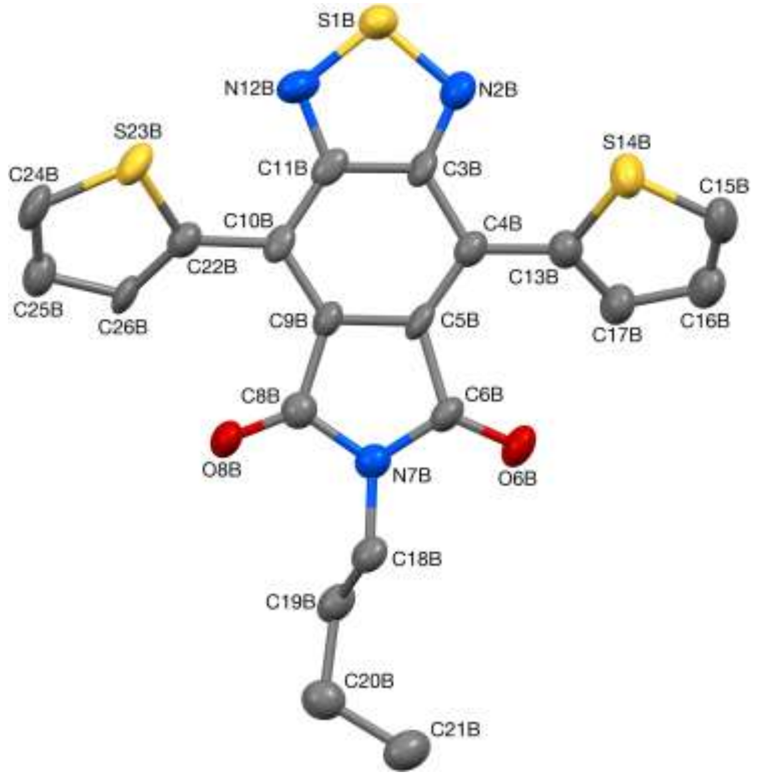

Figure S23. The structure of one (**5a-B**) of the two independent molecules present in the crystal of **5a** (50% probability ellipsoids).

(1) Tanaka, S.; Yamashita, Y. *Synth. Met.* **1995**, *69*, 599.
(2) Schroeder, B. C.; Nielsen, C. B.; Kim, Y. J.; Smith, J.; Huang, Z.; Durrant, J.; Watkins, S. E.; Song, K.; Anthopoulos, T. D.; McCulloch, I. *Chem. Mater.* **2011**, *23*, 4025.
(3) Nielsen, C. B.; Fraser, J. M.; Schroeder, B. C.; Du, J.; White, A. J. P.; Zhang, W.; McCulloch, I. *Org. Lett.* **2011**, *13*, 2414.
(4) Nielsen, C. B.; Ashraf, R. S.; Schroeder, B. C.; D'Angelo, P.; Watkins, S. E.; Song, K.; Anthopoulos, T. D.; McCulloch, I. *Chem. Commun.* **2012**, *48*, 5832.
(5) SHELXTL, Bruker AXS, Madison, WI; SHELX-97, G.M. Sheldrick, *Acta Cryst.*, **2008**, *A64*, 112-122; SHELX-2013, http://shelx.uni-ac.gwdg.de/SHELX/index.php
